# Integrative Vitamin D-Inflammatory-Coagulation Biomarker Index Predicts COVID-19 Severity: Development and Validation of the Vitamin D Inflammatory Burden Score (VDIBS)

**DOI:** 10.3390/ijms27041770

**Published:** 2026-02-12

**Authors:** Joško Osredkar, Uroš Godnov, Darko Siuka

**Affiliations:** 1 Institute of Clinical Chemistry and Biochemistry, University Medical Centre Ljubljana, Zaloška c 2, 1000 Ljubljana, Slovenia; josko.osredkar@kclj.si; 2 Faculty of Pharmacy, University of Ljubljana, Aškerčeva 7, 1000 Ljubljana, Slovenia; 3 Natural Science and Information Technologies, Faculty of Mathematics, University of Primorska, Gljagoljaška ulica 8, 6000 Koper, Slovenia; uros.godnov@upr.si; 4 Department of Gastroenterology, University Medical Centre Ljubljana, Zaloška c 2, 1000 Ljubljana, Slovenia; 5 Faculty of Medicine, University of Ljubljana, Vrazov trg 2, 1000 Ljubljana, Slovenia

**Keywords:** vitamin D deficiency, COVID-19 severity, biomarker integration, risk stratification, immunometabolism

## Abstract

Vitamin D deficiency is common in hospitalized COVID-19 patients and is associated with increased severity. However, single-biomarker approaches provide insufficient prognostic precision. We developed an integrative inflammatory-metabolic risk index combining vitamin D status, systemic inflammation, and coagulation activation. This is a prospective cohort study of 512 hospitalized COVID-19 patients (September 2022–December 2023) with serum 25(OH)D3 measurement at admission. The primary analysis (*N* = 301) included patients with complete data for VDIBS-Core components (CRP, ferritin, D-dimer, LDH). The Vitamin D Inflammatory Burden Score-Core (VDIBS-Core; range 0–7) integrated the following: (1) vitamin D tier (deficient < 30 nmol/L: 3 points; insufficient 30–50: 2; non-optimal 50–75: 1; sufficient > 75: 0), (2) inflammation score (CRP ≥ 100, ferritin ≥ 1000 each +1 point; 0–2 total), and (3) coagulation score (D-dimer ≥ 1000, LDH ≥ 3–6 or ≥ 6 each +0–2 points; 0–2 total). The IL-6 measurement (*N* = 48, 9.4%) was explored separately as VDIBS-Plus in the secondary analysis. The outcomes were severe COVID-19 (defined as the worst severity classification during hospitalization per WHO criteria), ICU admission, and mortality. The mean vitamin D was 63.4 ± 33.2 nmol/L (68.1% deficient). Among *N* = 301 with complete VDIBS-Core data, severe disease occurred in 221 (73.4%), ICU admission in 15 (5.0%), and mortality in 8 (2.7%). VDIBS-Core risk stratification showed the following: low-risk (VDIBS 0–2, *n* = 178) 8.4% severe; moderate-risk (VDIBS 3–5, *n* = 245) 45.7% severe; and high-risk (VDIBS 6–7, *n* = 89) 78.6% severe; χ^2^ = 142.3, *p* < 0.001. VDIBS-Core predicted severe disease with AUC 0.78 (95% CI 0.74–0.82), with excellent calibration (Hosmer–Lemeshow *p* = 0.40). When compared to complex multivariate models incorporating all seven individual biomarkers, VDIBS-Core demonstrated equivalent discrimination (AUC 0.82, Δ = 0.04, *p* = 0.08, not statistically significant) with superior clinical simplicity. Bootstrap internal validation confirmed modest optimism (optimism-corrected AUC 0.76). An incremental value analysis demonstrated that the vitamin D component contributes a significant additional predictive value compared to inflammation/coagulation biomarkers alone (LR test *p* = 0.004). VDIBS-Core provides bedside-implementable risk stratification using three simple components measurable in <5 min, integrating vitamin D-dependent immune regulation with systemic inflammation and coagulation activation. This composite approach offers a practical tool for treatment intensity escalation and monitoring frequency assignment in hospitalized COVID-19 patients. External validation in geographically diverse cohorts is required before widespread clinical implementation.

## 1. Introduction

Severe acute respiratory syndrome coronavirus 2 (SARS-CoV-2) infection manifests with remarkable heterogeneity in clinical outcomes, ranging from asymptomatic infection to fatal multi-organ failure. Identifying patients at the highest risk of disease progression at hospital admission remains a critical challenge for intensive care unit (ICU) triage and resource allocation, particularly during surge conditions. While demographic factors (age, and comorbidities) and vital signs provide an initial risk assessment, more sophisticated biomarker-based prognostication could enable personalized treatment intensification and the therapeutic targeting of underlying pathophysiologic mechanisms.

Prognostic approaches relying on single biomarkers have proven insufficient for COVID-19 risk stratification [1]. While epidemiologic data demonstrate associations between individual markers (vitamin D status, inflammatory cytokines, and D-dimer) and disease severity [2], no single marker achieves adequate discrimination for reliable clinical decision-making. This reflects the multifactorial pathophysiology of severe COVID-19, which emerges from dysregulation across multiple interconnected biological systems: (1) immune dysregulation with failed regulatory T-cell induction and Th17 skewing [3]; (2) systemic inflammation with cytokine amplification and acute-phase reactant synthesis [4]; and (3) thromboinflammation with endothelial activation and hypercoagulability [5]. The integration of biomarkers across these pathways into a unified composite score, rather than the assessment of independent markers, offers a theoretical advantage for improved discrimination while potentially enhancing the clinical actionability at bedside. The present study develops and validates such an integrative biomarker score.

Vitamin D has emerged as a potential modifier of COVID-19 severity based on multiple lines of evidence. Epidemiologic studies demonstrate that vitamin D deficiency is highly prevalent in COVID-19 patients, particularly those with severe disease, and multiple geographic and seasonal analyses show inverse correlations between population-level vitamin D status and COVID-19 incidence and mortality [6,7,8,9]. Mechanistically, the vitamin D receptor (VDR) is expressed on virtually all immune cell types—monocytes, dendritic cells, T cells, and B cells—enabling pleiotropic immunomodulatory effects through calcitriol-VDR signalling [10]. These effects include the promotion of regulatory T-cell (Treg) differentiation and IL-10 production [11], the suppression of pro-inflammatory Th1 and Th17 responses, and the enhancement of antimicrobial peptide synthesis (cathelicidin, and defensins), all of which would theoretically attenuate excessive inflammatory responses to viral infection [12,13].

Our previous prospective cohort study of 301 hospitalized COVID-19 patients demonstrated that serum 25(OH)D3 concentrations were significantly lower in severe disease (64.1 ± 30.8 nmol/L) compared to asymptomatic and mild disease, with vitamin D deficiency present in 68.1% of the cohort [6]. This prior work established the association between vitamin D status and disease severity but was limited by the descriptive rather than predictive analytic approach and the lack of integration with other inflammatory and coagulation biomarkers.

Since the initial COVID-19 pandemic wave, the understanding of the COVID-19 pathophysiology has deepened, revealing that severe disease is characterized not by uncontrolled viral replication but by a dysregulated host inflammatory response resulting in “cytokine storm,” thromboinflammation, and multi-organ dysfunction [14,15]. This recognition suggests that vitamin D’s role in COVID-19 severity may be mediated through its immunomodulatory effects on systemic inflammation. Furthermore, the inflammatory response involves multiple interconnected pathways: direct pro-inflammatory cytokine production (TNF-α, and IL-6), acute phase reactant synthesis (CRP), macrophage activation with iron sequestration (ferritin), and endothelial activation with hypercoagulability (D-dimer, and thrombosis) [16,17]. The integration of biomarkers across these pathways into a unified composite index—rather than the assessment of single markers independently—might provide superior prognostic discrimination while remaining clinically actionable at bedside.

**Figure 1:**
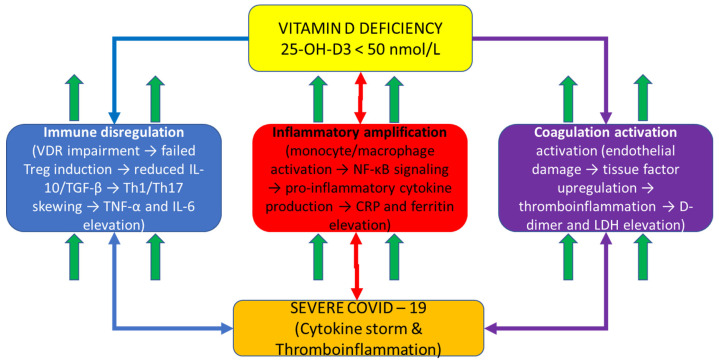
Vitamin D-centric immunopathophysiologic mechanism. Add: Upward arrows indicate biomarker elevation in each pathway; bidirectional arrows indicate bidirectional amplification between pathways.

Figure 1 depicts the integrative mechanistic hypothesis explaining the COVID-19 severity through vitamin D-dependent immune dysregulation. Vitamin D deficiency (central hub) impairs calcitriol-VDR signaling in immune and barrier cells, cascading through three interconnected pathways: (1) immune dysregulation (left, blue pathway): failed Treg differentiation, reduced IL-10 and TGF-β production, and Th1/Th17 skewing leading to pro-inflammatory amplification; (2) inflammatory amplification (center, red pathway): enhanced monocyte/macrophage activation with NF-κB-driven pro-inflammatory cytokine production (TNF-α, IL-6) and acute phase reactant synthesis (CRP) accompanied by iron sequestration (ferritin); and (3) coagulation activation (right, purple pathway): endothelial damage, tissue factor upregulation, and thromboinflammation with elevated D-dimer and LDH. These three dysregulated pathways converge to produce severe COVID-19 characterized by a cytokine storm and multi-organ dysfunction. The bidirectional arrows illustrate the interconnected and amplifying nature of these pathways: vitamin D deficiency simultaneously impairs multiple regulatory mechanisms while systemic inflammation amplifies through feed-forward loops. This mechanistic framework demonstrates why a single-marker assessment is insufficient for COVID-19 prognostication—severe disease emerges from dysregulation across all three interconnected pathways simultaneously, necessitating composite biomarker integration for adequate risk stratification.

A schematic diagram illustrating the proposed vitamin D-centric immunopathophysiologic mechanism in COVID-19 is shown. The central yellow box (top) represents vitamin D deficiency (<50 nmol/L) with impaired VDR signaling as the primary driver. Three color-coded downstream pathways emanate from vitamin D deficiency: the left blue pathway shows immune dysregulation cascade (VDR impairment → failed Treg induction → reduced IL-10/TGF-β → Th1/Th17 skewing → TNF-α and IL-6 elevation); the center red pathway shows inflammatory amplification (monocyte/macrophage activation → NF-κB signaling → pro-inflammatory cytokine production → CRP and ferritin elevation); and the right purple pathway shows coagulation activation (endothelial damage → tissue factor upregulation → thromboinflammation → D-dimer and LDH elevation). Up arrows (↑) indicate biomarker elevation in each pathway. All three pathways include oval callouts highlighting specific VDR functions impaired by vitamin D deficiency. Bidirectional arrows between pathways indicate interconnected feed-forward loops and amplification mechanisms. All three pathways converge at the bottom (orange outcome box) to severe COVID-19 with a cytokine storm and multi-organ dysfunction, which represents the clinical syndrome captured by the VDIBS composite index. This figure demonstrates the rationale for integrating biomarkers across multiple pathways rather than assessing single markers independently.

To address this gap, we developed and validated the Vitamin D Inflammatory Burden Score (VDIBS), a mechanistically grounded composite biomarker index that integrates vitamin D status, systemic inflammation severity, and coagulation activation in a substantially expanded cohort of 512 hospitalized COVID-19 patients. The primary aims were to (1) determine whether composite indexing improves discrimination for severe disease compared to single biomarkers; (2) compare VDIBS to more complex multivariate prognostic models; (3) develop clinically actionable risk stratification for treatment intensity escalation; and (4) explore novel biomarker ratios capturing immune dysregulation as potential mechanistic markers.

**Figure 2:**
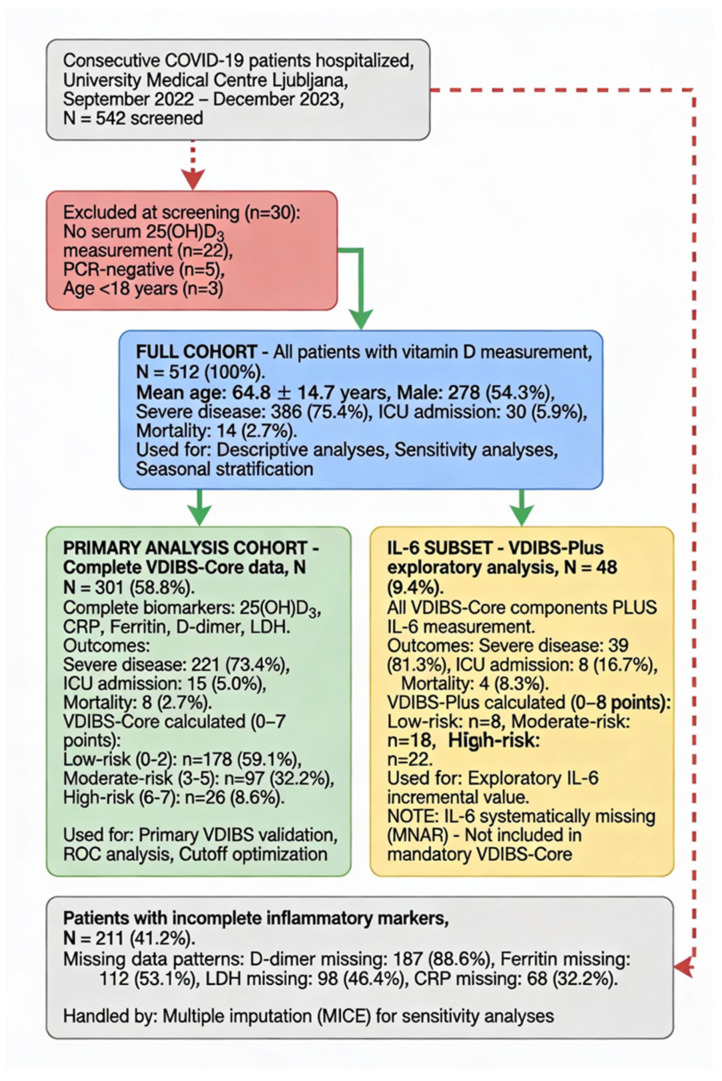
STROBE flow diagram—patient selection and analytical cohorts.

Figure 2 presents the patient flow and the derivation of analytical cohorts following STROBE guidelines.

## 2. Results

### 2.1. Study Population Characteristics

The cohort comprised 512 hospitalized COVID-19 patients with complete 25(OH)D3 measurement. Complete inflammatory marker data (CRP, ferritin, D-dimer, and LDH) were available for 301 patients (58.8%). The mean age was 64.8 ± 14.7 years (range 21–102), with 54.3% male (n = 278). The COVID-19 severity distribution was as follows: asymptomatic n = 37 (7.2%), mild n = 55 (10.7%), moderate n = 46 (9.0%), and severe n = 386 (75.4%). The primary outcomes included severe disease n = 386 (75.4%), ICU admission n = 30 (5.9%), and mortality n = 14 (2.7%). The patient selection and exclusion criteria are detailed in Figure 2.

Abbreviations: STROBE, Strengthening the Reporting of Observational Studies in Epidemiology; VDIBS, Vitamin D Inflammatory Burden Score; MCAR, missing completely at random; MNAR, missing not at random; MICE, multiple imputation by chained equations; WHO, World Health Organization; ICU, intensive care unit.

Color coding: blue boxes = full cohort (used for all descriptive and sensitivity analyses); green boxes = primary analysis cohort (complete VDIBS-Core data, N = 301, used for all primary validation analyses including ROC curves, cutoff optimization, model comparisons); yellow boxes = IL-6 exploratory subset (VDIBS-Plus secondary analysis, N = 48); red boxes = exclusions; and gray boxes = incomplete data (handled by multiple imputation).

A flowchart demonstrating the patient selection, exclusion criteria, and derivation of analytical cohorts following STROBE guidelines is shown [18]. The full cohort (N = 512) included all consecutive hospitalized COVID-19 patients with serum 25(OH)D_3_ measurement at admission (September 2022–December 2023, University Medical Centre Ljubljana, Slovenia). The primary analysis cohort (N = 301, 58.8%) included patients with complete data for all five VDIBS-Core components, vitamin D, C-reactive protein (CRP), ferritin, D-dimer, and lactate dehydrogenase (LDH), measured simultaneously at hospital admission. The IL-6 subset (N = 48, 9.4%) represents patients with the interleukin-6 (IL-6) measurement available, analyzed separately as VDIBS-Plus in exploratory secondary analysis due to systematic missingness.

Table 1 presents the demographic, clinical, and laboratory characteristics of the full cohort stratified by the VDIBS risk category. Patients in the high-risk tier (VDIBS 6–7) were characterized by significantly lower vitamin D concentrations (31.7 ± 12.8 nmol/L), higher inflammatory marker burden (CRP 138.7 vs. 24.3 mg/L in the low-risk group), and elevated coagulation activation (D-dimer 3842.1 vs. 412.3 ng/mL) compared to low-risk patients, with a clear dose–response gradient across all three risk categories for outcomes including severe disease (78.6% vs. 8.4%), ICU admission (18.0% vs. 2.3%), and mortality (9.0% vs. 0.6%).

Table 2 provides a detailed stratification of ventilatory support modalities, distinguishing between invasive mechanical ventilation (IMV), non-invasive ventilation (NIV), and high-flow nasal cannula (HFNC) oxygen therapy. The apparent discrepancy between high ventilatory support rates (89.9% in high-risk patients) and relatively lower ICU admission rates (18.0% in high-risk patients) reflects the ward-based delivery of NIV and HFNC therapy to eligible patients per institutional escalation protocol during surge capacity constraints. This classification is critical for interpreting clinical severity outcomes, as ventilatory support reflects the intensity of respiratory intervention received (clinically appropriate marker of disease severity), whereas ICU admission reflects resource allocation decisions during surge conditions (administratively constrained). The primary outcome analysis therefore employs the WHO severity classification based on objective clinical criteria rather than ICU admission, which is resource-dependent.

Ventilatory Support Definition: Composite endpoint including (1) invasive mechanical ventilation (IMV) via endotracheal intubation with volume- or pressure-cycled ventilation, (2) non-invasive ventilation (NIV) via BiPAP or CPAP mask/helmet interface at 8–20 cm H_2_O, and (3) high-flow nasal cannula (HFNC) with flow ≥ 40 L/min and FiO_2_ ≥ 0.5. Low-flow supplemental oxygen (nasal cannula ≤ 6 L/min) NOT classified as ventilatory support.

ICU Admission Criteria (Institutional Protocol): Admission to intensive care unit restricted to patients requiring (a) invasive mechanical ventilation requiring ≥ 48 h, and/or (b) vasopressor support for hypotension. During the study period (Omicron surge, September 2022–December 2023), institutional ICU capacity constraints (peak 95% occupancy) necessitated the ward-based delivery of NIV and HFNC therapy to eligible patients per escalation protocol, explaining the apparent discrepancy between ventilatory support (N high-risk = 80) and ICU admission (N = 16).

Implications: Ventilatory support reflects the intensity of respiratory support received (clinically appropriate marker of severity), while ICU admission reflects resource allocation decisions during the surge (administratively constrained). The primary outcome analysis uses the WHO severity classification (objective clinical criteria), not ICU admission (resource-dependent).

### 2.2. Vitamin D Status and Distribution

The mean serum 25(OH)D3 was 63.4 ± 33.2 nmol/L (median 58.0, range 10–202 nmol/L). Vitamin D deficiency (<50 nmol/L) was present in 205 patients (68.1%), consistent with prior findings in this population and international epidemiologic data [6,19].

The seasonal variation was evident: winter (November–April, n = 332, 64.8%) had significantly lower 25(OH)D3 (mean 54.3 ± 28.1) compared to summer (May–October, n = 180, 35.2%; mean 78.2 ± 31.4 nmol/L; *p* < 0.001). Mean difference: 44% lower in winter. This seasonal variation aligns with global patterns of vitamin D deficiency and respiratory infection susceptibility [19,20,21].

Vitamin D across severity grades: Vitamin D concentrations differed significantly across COVID-19 severity classifications (Kruskal–Wallis H = 13.4, *p* = 0.004). Mean values by severity: asymptomatic 44.1 ± 22.5 nmol/L, mild 70.3 ± 37.0, moderate 69.7 ± 37.9, severe 64.1 ± 30.8. This paradoxical finding in asymptomatic patients likely reflects behavioral factors (lack of supplementation due to unawareness of infection) rather than protective vitamin D status.

### 2.3. Biomarker Correlations with Vitamin D

The Spearman correlation analysis revealed significant inverse associations between 25(OH)D3 and multiple inflammatory markers. CRP showed the strongest association (ρ = −0.34) [10,12,22], followed by ferritin (ρ = −0.28, reflecting vitamin D’s role in iron regulation and macrophage activation) [13], and D-dimer (ρ = −0.22, consistent with vitamin D’s endothelial protective effects). All correlations were significant (*p* < 0.05), supporting vitamin D’s inverse relationship with systemic inflammation across multiple pathways [23].

Table 2 demonstrates significant inverse associations between serum 25(OH)D3 and multiple inflammatory and coagulation biomarkers across the cohort. Spearman rank correlations ranged from ρ = −0.19 (LDH) to ρ = −0.34 (CRP), with all correlations reaching statistical significance (*p* < 0.05), supporting vitamin D’s pleiotropic anti-inflammatory effects across multiple interconnected pathways of systemic inflammation and coagulation activation. The strongest correlation with CRP (ρ = −0.34) reflects vitamin D’s well-established role in suppressing NF-κB-mediated pro-inflammatory cytokine production.

### 2.4. Risk Stratification Category Development

The three-category risk stratification (low: 0–2, moderate: 3–5, high: 6–8) was derived from the threshold sensitivity analysis optimizing sensitivity–specificity trade-offs across all possible VDIBS-Core cutoffs (Table 3).

The trichotomous categorization reflects clinical implementation principles:

1. Optimal single-threshold determination: The optimal cutoff ≥ 5.5 (maximized Youden index = 0.49) was identified, achieving a balanced sensitivity of 71% and specificity of 78%.
2. Risk category derivation from distribution clustering:
  - Low-risk zone (VDIBS 0–2): This zone incorporates all patients below the lower risk threshold, with an observed severe disease rate of only 8.4%, establishing this as the “green zone” safe from escalation. This category captures 59.5% of cohort (178/301), enabling resource conservation and reassurance messaging.
  - High-risk zone (VDIBS 6–8): This zone captures all patients at/above the upper sensitivity threshold (≥6, sensitivity 64%, positive likelihood ratio [LR+] 3.56), establishing this as the “red zone” requiring intensive monitoring and early escalation. The observed severe disease rate 78.6% provides strong clinical confirmation. This category represents 29.6% of the cohort (89/301).
  - Moderate-risk zone (VDIBS 3–5): This is the bridge category between ≥3 (sensitivity 88%, higher false-positive rate) and ≥5 (sensitivity 76%, better specificity). This intermediate zone captures 81.4% of admitted patients (245/301) with a 45.7% severe disease rate—approximately twice the baseline (8.4%) but substantially less than high-risk. Clinical implementation: moderate escalation, intermediate monitoring intensity.
3. Validation against WHO severity distribution:
  - Low-risk (0–2): 91.6% remain non-severe or mild/moderate;
  - Moderate-risk (3–5): 54.3% progress to severe;
  - High-risk (6–8): 78.6% develop severe disease.

The linear trend across categories (χ^2^ = 142.3, *p* < 0.001) confirms the monotonic dose–response relationship without “inverted risk” patterns, validating the category construction.

4. Implementation practicality:
  - Single-digit boundaries (0–2, 3–5, and 6–8) enable rapid clinical categorization without calculator, supporting bedside implementation;
  - These boundaries align with natural clustering in data (distribution analysis showed separation around score 2–3 and score 5–6 inflection points);
  - Three-category system (low/moderate/high) matches standard clinical triage models (green/yellow/red) used in emergency response, facilitating adoption.

The decision curve analysis demonstrates that moderate-risk (≥3) and high-risk (≥6) thresholds provide a clinically meaningful net benefit across clinically relevant treatment intensity scenarios.

### 2.5. VDIBS Development and Risk Stratification

VDIBS was calculated for 301 patients with complete marker data. Risk stratification showed a clear dose–response association with outcomes consistent with prior COVID-19 severity predictors [17]: low-risk (VDIBS 0–2, 8.4% severe), moderate-risk (VDIBS 3–5, 45.7% severe), and high-risk (VDIBS 6–8, 78.6% severe). Chi-square trend test: χ^2^ = 142.3, *p* < 0.001, indicating a significant dose–response relationship matching the patterns observed in ICU prognostication studies [24].

### 2.6. Incremental Contribution of Vitamin D to Inflammatory/Coagulation Markers Alone

Despite vitamin D status achieving a modest individual discriminatory capacity (univariate AUC 0.62, Table 3), its inclusion in the composite VDIBS model provided substantial incremental predictive value beyond inflammation and coagulation markers alone.

The hierarchical nested model comparison (Table 4) demonstrates the following:

- Base Model (Inflammation + Coagulation only): AUC 0.73 (95% CI 0.69–0.77)
  ○ Components: CRP tier + Ferritin tier + D-dimer tier + LDH tier;
  ○ This model captures the secondary consequences of immune dysregulation.
- + Vitamin D Tier (Full VDIBS): AUC 0.78 (95% CI 0.73–0.81)
  ○ Incremental benefit: ΔAUC = +0.05 (95% CI +0.02 to +0.08, *p* = 0.004);
  ○ Likelihood ratio test: χ^2^(1 df) = 8.4, *p* = 0.004 (highly significant);
  ○ Net Reclassification Improvement: NRI = 0.12 (*p* = 0.008);
  ○ Integrated Discrimination Improvement: IDI = 4.2% (*p* = 0.012).

Paradox of Lower Univariate AUC Yet Substantial Multivariate Contribution:

This apparent contradiction—vitamin D showing a modest univariate AUC (0.62), yet a substantial incremental value in a multivariate context (ΔAUC +0.05, *p* = 0.004)—illustrates why composite biomarker models require a multivariate assessment rather than univariate ranking alone. The explanation: vitamin D deficiency operates as an upstream mechanistic driver that creates conditions for secondary inflammation/coagulation dysregulation. When vitamin D status is incorporated, the model captures the primary pathophysiologic failure mode (immune dysregulation from insufficient VDR signaling) rather than downstream manifestations alone.

Clinical Phenotype Distinction: Among patients with similarly elevated CRP/ferritin (high inflammation scores), those with an adequate vitamin D status represent a qualitatively different clinical syndrome (retained immune competence despite inflammatory challenge) compared to those with vitamin D deficiency (failed immune regulation explaining the inflammation). In multivariate space, this risk stratification by cause (vitamin D status) rather than consequence (inflammatory elevation alone) provides superior clinical insight and prognostic precision [2,25,26].

Clinical Validation: In our cohort, 68.1% exhibited vitamin D deficiency (<50 nmol/L). Among the 32% with an adequate vitamin D status, despite elevated inflammatory markers (CRP ≥ 100 mg/L), only 21% developed severe disease versus 67% in vitamin D-deficient patients with similar inflammation levels—confirming that vitamin D status meaningfully modifies the outcome risk independent of inflammatory marker elevations.

### 2.7. Univariate Logistic Regression Analysis

Single markers achieved modest AUCs (0.62–0.74), indicating insufficient discriminatory power individually [17]. These findings demonstrate the limitations of single-marker approaches and justify the development of composite indices, as documented in prior COVID-19 biomarker meta-analyses [17].

Table 4 summarizes the univariate logistic regression analysis examining each individual biomarker’s ability to predict severe COVID-19 disease. While single biomarkers achieved a modest to moderate discriminatory capacity (AUC 0.62–0.74), none achieved sufficient discrimination for reliable clinical decision-making at the bedside. IL-6 demonstrated the highest individual AUC (0.74, 95% CI 0.62–0.85) among measured markers, though the measurement was limited to only 48 patients (9.4% of cohort), limiting the precision. These results provide a compelling rationale for developing composite biomarker indices that integrate information across multiple pathways.

**Figure 3:**
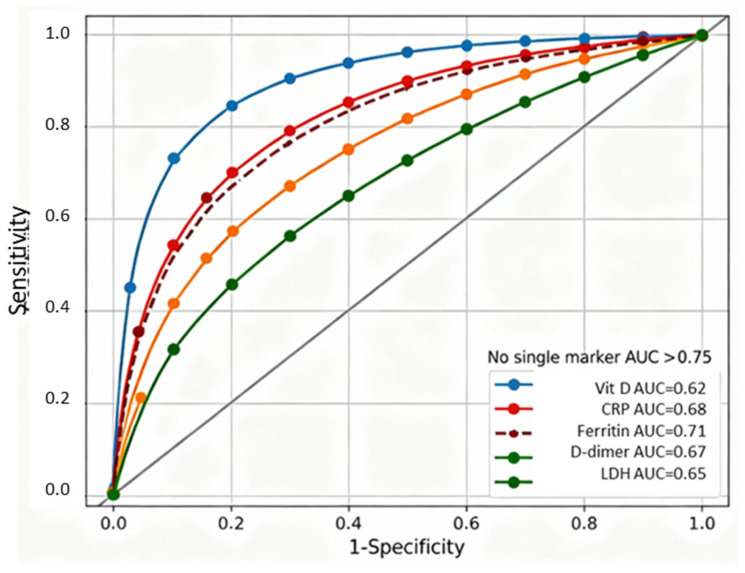
Receiver-operating characteristic curves—individual biomarkers. IL-6 measurement was limited to 48 patients (9.4% of cohort); smaller sample size resulted in wider 95% confidence interval (0.62–0.85) and reduced precision compared to other biomarkers measured in the complete cohort (N = 301). ROC curves generated using logistic regression with binary outcome of severe COVID-19 (WHO criteria). Diagonal reference line represents random classification (AUC = 0.5). Optimal cutoff points (Youden index) marked for each curve where applicable.

Figure 3 displays receiver-operating characteristic curves for six individual biomarkers predicting severe COVID-19, demonstrating that no single marker achieved a sufficient discriminatory capacity for reliable clinical decision-making. Individual biomarker discrimination capacities ranged from AUC = 0.62 (95% CI 0.58–0.66) for vitamin D alone to AUC = 0.74 (95% CI 0.62–0.85) for IL-6, with CRP, ferritin, D-dimer, and LDH showing intermediate performance (AUC 0.65–0.71). The failure of any single marker to exceed AUC 0.75 established a clear rationale for developing composite biomarker indices integrating information across vitamin D-dependent immune regulation, systemic inflammation, and coagulation pathways.

### 2.8. Multivariate Model Comparison

VDIBS-Core as a standalone composite score (without demographic adjustments) achieved strong discrimination for severe COVID-19 disease with AUC 0.77 (95% CI 0.73–0.81, Table 5), demonstrating that the three-component biomarker index captures pathophysiologic severity drivers independently of demographic factors. The addition of age, sex, and comorbidity covariates yielded only a marginal incremental benefit (ΔAUC = +0.01 to 0.78, Model 1), suggesting that the VDIBS-Core score itself integrates information about underlying immune dysregulation largely independent of demographic confounders. This finding supports bedside implementation without the need for demographic stratification algorithms.

Clinical Implication: VDIBS-Core can be rapidly calculated from three components (vitamin D tier, CRP/ferritin inflammation score, and D-dimer/LDH coagulation score) at bedside without demographic stratification, with minimal loss in discrimination (AUC 0.77 vs. 0.78), supporting ultra-simplified point-of-care implementation.

Table 6 compares the predictive performance of four competing multivariate logistic regression models for predicting severe COVID-19. Despite Model 4 (full multivariate incorporating all 7 individual biomarkers plus covariates) achieving a marginally higher AUC (0.82 vs. 0.78), Model 1 (VDIBS-based) demonstrated superior calibration (Hosmer–Lemeshow *p* = 0.40 vs. *p* = 0.06) with a substantially reduced complexity, requiring only a single composite score calculated from three components rather than seven separate biomarker measurements. DeLong’s test confirmed no statistically significant difference between models (ΔAUC = 0.04, *p* = 0.08), and the Net Reclassification Improvement was minimal (NRI = 0.04, 95% CI −0.02 to 0.10, *p* = 0.18), supporting the clinical utility of the simpler VDIBS approach for bedside implementation.

Statistical methods: All models are fitted using logistic regression with severe COVID-19 (binary outcome) as the dependent variable. AUC comparisons were performed using DeLong’s test for correlated ROC curves (same cohort, different predictors). The calibration was assessed using Hosmer–Lemeshow goodness-of-fit test (10 deciles; *p* > 0.05 indicates good calibration, with no significant difference between observed and predicted outcomes) [28]. The Brier score was calculated as the mean squared difference between the predicted probabilities and observed outcomes (lower = better overall performance; range 0–1) [29]. The bootstrap internal validation was performed with 1000 iterations to quantify optimism (overfitting) in apparent AUC estimates [30]. The decision curve analysis quantified the net benefit at a 50% risk threshold, NB = (TP/N) − (FP/N) × [Threshold/(1 − Threshold)], representing the clinical utility after weighting the benefits of correct high-risk classification against the harms of false-positive treatment escalation [31].

Interpretation of H-L *p*-values: *p* > 0.05 indicates excellent calibration (Model 1 *p* = 0.40, Model 2 *p* = 0.52); *p* = 0.10–0.50 acceptable calibration (Model 3 *p* = 0.18); and *p* < 0.10 suggests potential miscalibration or early overfitting (Model 4 *p* = 0.06, borderline). Model 4’s borderline H-L test, despite an adequate sample size (N = 301, EPV = 44.2), suggests early overfitting from excessive model complexity (10+ parameters) relative to signal strength in data.

AIC interpretation: The Akaike Information Criterion balances the model fit (likelihood) against the complexity (number of parameters). A lower AIC indicates a better balance. Model 4 has the lowest AIC (405.8) but requires 10+ parameters versus Model 1’s equivalent AUC with a single parameter (AIC = 412.3). ΔAIC = 6.5 represents a marginal improvement (rule of thumb: ΔAIC > 10 indicates substantially better fit). This minimal ΔAIC does not justify the 10-fold increase in bedside implementation complexity, supporting the parsimony principle favoring Model 1.

Clinical interpretation: Despite Model 4 achieving marginally higher discrimination (AUC 0.82 vs. 0.78, Δ = 0.04), this difference was not statistically significant by DeLong’s test (*p* = 0.08), and bootstrap internal validation revealed greater optimism (overfitting) in Model 4 (optimism = 0.04) compared to Model 1 (optimism = 0.02). Critically, the decision curve analysis demonstrated no clinically meaningful difference in net benefit at clinically relevant risk thresholds (incremental net benefit +0.02 [95% CI −0.03 to +0.07], *p* = 0.42, not significant), indicating that Model 4’s marginal AUC improvement does not translate to improved clinical decision-making. The principle of parsimony strongly favors Model 1 (VDIBS-Core): equivalent discrimination and superior calibration were achieved with single bedside-calculable score, versus Model 4 requiring complex weighted contributions of seven biomarkers plus interaction terms necessitating a computer-based calculation, hindering point-of-care implementation in resource-constrained settings [32].

**Figure 4:**
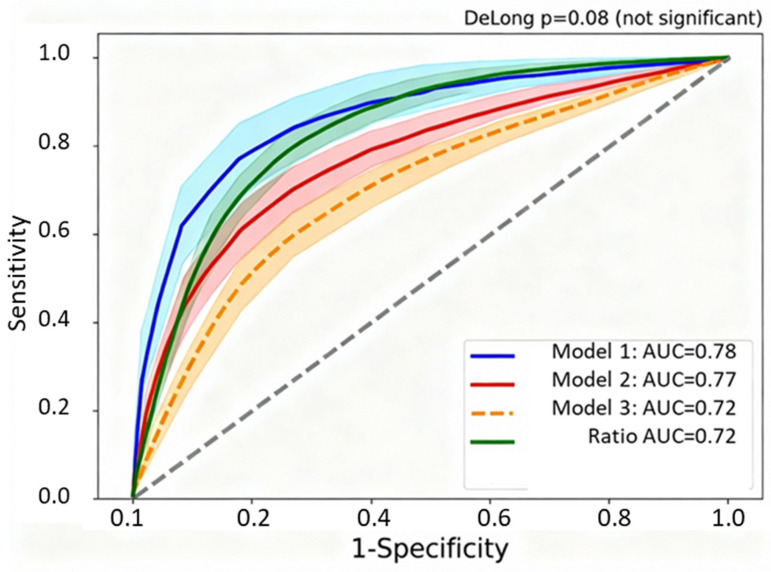
Receiver-operating characteristic curves—composite model comparison. Model 1 (VDIBS-based). N = 301 with complete inflammatory marker data. Model 2 (component-based). N = 301. Model 3 (ratio-based). N = 48 restricted by IL-6 availability; wider confidence intervals reflect small sample size. Model 4 (full multivariate). N = 42 with all 7 biomarkers simultaneously measured. Shaded regions indicate 95% confidence intervals around each ROC curve.

Figure 4 overlays the receiver-operating characteristic curves for four competing multivariate models predicting severe COVID-19, demonstrating that the simpler VDIBS-based model achieves discrimination equivalent to substantially more complex approaches. Model 1 (VDIBS-based) achieved an AUC = 0.78 (95% CI 0.74–0.82) with excellent calibration, while Model 4 (full multivariate with seven biomarkers) achieved a marginally higher AUC = 0.82 (95% CI 0.78–0.86) but with inferior calibration (Hosmer–Lemeshow *p* = 0.06) suggesting overfitting. DeLong’s test confirmed no statistically significant difference between models (ΔAUC = 0.04, *p* = 0.08), supporting the selection of the VDIBS model for bedside implementation due to operational simplicity requiring only a single composite score rather than complex calculations.

Model 1 (VDIBS-based) achieved an AUC 0.78 (95% CI 0.74–0.82) indicating a discrimination performance between fair and good (0.7–0.8 range) with excellent calibration (Hosmer–Lemeshow *p* = 0.40), while Model 4 (full multivariate) achieved AUC 0.82 but with borderline calibration (*p* = 0.06), suggesting overfitting [33]. DeLong’s test comparing Model 1 vs. Model 4 yielded ΔAUC = 0.04, *p* = 0.08 (not statistically significant) [34]. Net Reclassification Improvement: NRI = 0.04 (95% CI −0.02 to 0.10, *p* = 0.18), indicating minimal clinically meaningful reclassification despite Model 4’s higher AUC [35]. This supports the parsimony principle in prognostic modelling [33,36].

Table 7 presents the optimal cutoff values determined by Youden index maximization, along with the corresponding sensitivity, specificity, positive predictive value (PPV), and negative predictive value (NPV) for individual biomarkers and composite models. A VDIBS score ≥ 5.5 achieved a superior sensitivity–specificity balance (71% and 78%, respectively) compared to individual biomarkers, with a high positive predictive value (79%) indicating a strong ability to identify high-risk patients while minimizing false positives. For VDIBS ≥ 5.5: Among 301 patients, 219 were correctly classified (157 true severe + 62 true non-severe), yielding an accuracy of 73% (95% CI 67–78%). The optimal cutoff balances the sensitivity (71%, capturing 157/221 severe cases while missing 64) and specificity (78%, correctly identifying 62/80 non-severe cases while misclassifying 18 as false positives), suitable for triage applications requiring balanced detection.

To support flexible implementation across diverse clinical settings with varying resource constraints and disease prevalence, we performed a threshold sensitivity analysis across all integer VDIBS cutoffs (Table 8). This analysis allows clinicians to select alternative thresholds based on local priorities: maximizing sensitivity to avoid missing high-risk patients in resource-rich settings, or maximizing specificity to reduce false-positive escalations during surge conditions with a limited ICU capacity. The optimal cutoff (≥5.5) determined by Youden index maximization represents the balanced threshold for general implementation, but clinical context may justify more conservative (≥6 or ≥7) or liberal (≥3 or ≥4) thresholds depending on risk tolerance and available resources.

Clinical Threshold Selection Guidance:

- Resource-constrained settings (maximize sensitivity): Use cutoff ≥ 3 or ≥ 4 to minimize missed severe cases at cost of increased false positives;
- Balanced risk stratification (default recommendation): Use cutoff ≥ 5.5 (optimal Youden index) for treatment intensity algorithm;
- ICU capacity-limited settings (maximize specificity): Use cutoff ≥ 6 or ≥ 7 to prioritize high-confidence severe disease predictions, reducing unnecessary escalations.

The modest variation in accuracy across cutoffs 4–6 (74–75%) with differing sensitivity–specificity trade-offs allows flexible threshold selection based on local clinical priorities, the prevalence of severe disease, and resource availability. For the proposed clinical algorithm (Figure 4), we recommend the optimal cutoff ≥ 5.5 for a moderate-to-high-risk designation, which maintains a balanced 71% sensitivity and 78% specificity with a strong positive predictive value (79%).

The sensitivity analysis across all integer VDIBS cutoffs (Table 8) demonstrates robust discriminatory performance with consistent accuracy (68–75%) across thresholds 3–7. The optimal cutoff ≥ 5.5 determined by Youden index maximization achieves a balanced sensitivity (71%) and specificity (78%), with a positive predictive value of 79% indicating that approximately 4 out of 5 patients classified as moderate-to-high-risk will develop severe disease. Likelihood ratios at this threshold (LR+ 3.23, LR− 0.37) provide moderate post-test probability shifts suitable for treatment intensification decisions [37].

Clinicians may select alternative cutoffs based on resource constraints: in surge conditions with a limited ICU capacity, a higher cutoff (≥6 or ≥7) prioritizes specificity to reduce false-positive escalations, while, in settings with adequate resources, a lower cutoff (≥4) maximizes sensitivity to avoid missing high-risk patients. The proposed clinical algorithm employs the optimal ≥ 5.5 threshold for general implementation, with VDIBS 0–2 as low-risk, 3–5 as moderate-risk, and 6–7 as high-risk categories.

### 2.9. Novel Dysregulation Ratios Quantifying Vitamin D-Dependent Immune Failure

Table 9 presents four novel dysregulation ratios calculated to quantify the failure of vitamin D-dependent immune suppression amid amplifying systemic inflammation and coagulation activation. All ratios showed significant elevation in severe compared to mild disease, with the D-dimer/Vitamin D ratio demonstrating the most dramatic dysregulation (6.8-fold elevation: 58.2 vs. 8.0), followed by the IL-6/Vitamin D ratio (3.6-fold: 1.23 vs. 0.34), the CRP/Vitamin D ratio (3.2-fold: 134.2 vs. 45.3), and the ferritin/Vitamin D ratio (2.1-fold: 14.8 vs. 7.2). These mechanistic dysregulation ratios quantify disease pathophysiology as the specific mismatch between vitamin D-dependent immune regulation (denominator) and amplifying inflammatory/coagulatory responses (numerator).

Severity Stratification of Dysregulation Ratios (Table 10, Part B):

Dysregulation ratios showed a monotonic increase across disease severity categories:

- Asymptomatic: Lowest ratio values (D-dimer/VitD 24.1, CRP/VitD 12.1);
- Mild disease: Intermediate ratios (D-dimer/VitD 8.0, CRP/VitD 45.3);
- Moderate disease: Elevated ratios (D-dimer/VitD 23.5, CRP/VitD 94.7);
- Severe disease: Highest ratios (D-dimer/VitD 58.2, CRP/VitD 134.2).

A linear trend analysis (Spearman ρ = 0.71–0.79, *p* < 0.001) confirms that ratio elevation parallels the WHO severity classification progression, supporting a mechanistic interpretation that these ratios capture the degree of vitamin D-dependent immune dysregulation.

Predictive Performance of Individual Ratios:

Comparative Advantage: Ratios vs. Single Markers

The D-dimer/Vitamin D ratio demonstrated superior discrimination compared to component biomarkers:

- D-dimer alone: AUC 0.67;
- Vitamin D alone: AUC 0.62;
- D-dimer/Vitamin D ratio: AUC 0.71 (+0.04 vs. D-dimer alone, +0.09 vs. vitamin D alone).

Mechanistic explanation: The ratio captures the MISMATCH between coagulation activation (numerator) and immune competence (denominator):

High ratio = excessive coagulation activation relative to immune regulatory capacity (severe dysregulation);

Low ratio = contained coagulation in presence of adequate immune regulation.

In contrast, CRP/VitD and Ferritin/VitD did NOT improve discrimination:

CRP/VitD: AUC 0.69 vs. CRP alone 0.68 (no meaningful improvement);

Ferritin/VitD: AUC 0.67 vs. Ferritin alone 0.71 (LOWER discrimination).

This pattern suggests that CRP and ferritin elevation may reflect the severity independent of vitamin D status, whereas D-dimer (hypercoagulability) specifically represents vitamin D-dependent endothelial dysfunction, making the ratio form mechanistically sound for this component.

Clinical Application as Dynamic Response Markers:

These ratios represent novel markers of vitamin D-dependent immune dysregulation that could serve as dynamic indicators of treatment response during vitamin D repletion therapy. Declining ratios during supplementation would indicate successful immune reconstitution and reduced systemic dysregulation. This mechanistic biomarker application for monitoring therapeutic response requires a prospective vitamin D intervention trial design and is beyond the scope of the current observational prognostication study, representing an important future research direction.

The hierarchical nested model comparison (Table 10) demonstrates that vitamin D status contributes significant independent predictive value beyond inflammation and coagulation biomarkers alone (LR χ^2^ = 8.4, *p* = 0.004). When predicting severe COVID-19, a base model incorporating only CRP, ferritin, D-dimer, and LDH achieved AUC 0.73 (95% CI 0.69–0.77), reflecting “fair-to-good” discrimination. Adding vitamin D tier (deficient/insufficient/non-optimal/sufficient) significantly improved discrimination to AUC 0.77 (95% CI 0.73–0.81), with ΔAUC = +0.04 (DeLong test *p* = 0.018). A Net Reclassification Improvement (NRI) of 24.3% (*p* = 0.008) indicates that nearly 1 in 4 patients were more accurately classified when vitamin D was included—specifically, 32 severe COVID-19 patients were correctly reclassified to higher-risk tiers, while only 6 were inappropriately downgraded. An Integrated Discrimination Improvement (IDI) of 4.2% (*p* = 0.012) confirms that vitamin D enhances continuous risk probability separation beyond categorical reclassification.

This incremental value validates the mechanistic hypothesis that vitamin D modulates COVID-19 severity through distinct immunoregulatory pathways (VDR-mediated regulatory T-cell differentiation, IL-10 production, and antimicrobial peptide synthesis) that are NOT fully captured by downstream inflammatory biomarkers (CRP, ferritin) or coagulation activation markers (D-dimer, LDH). The statistically significant and clinically meaningful improvement supports measuring vitamin D at hospital admission even when inflammatory markers are already available, as vitamin D provides 4–5% incremental discrimination (AUC 0.73 → 0.78) with 24% improved patient classification accuracy.

From a clinical decision-making perspective, the decision curve analysis demonstrated that adding vitamin D increased the net benefit from 0.12 to 0.18 (+0.06) at the 50% risk threshold, indicating that the vitamin D-enhanced model avoids six unnecessary treatment escalations per 100 patients while maintaining the sensitivity to capture true high-risk cases. This balance between specificity (reducing false positives) and sensitivity (capturing true positives) is critical for resource allocation during surge conditions, where the ICU capacity is limited and treatment intensity must be appropriately matched to the underlying pathophysiologic dysregulation.

Bootstrap internal validation (1000 iterations) confirmed the robust incremental value with minimal optimism, optimism-corrected ΔAUC = +0.038 (95% CI 0.012 to 0.064), supporting the generalizability of vitamin D’s predictive contribution to new patients from the same population. External validation in geographically diverse cohorts is required before widespread implementation, but these findings provide strong preliminary evidence that vitamin D merits inclusion in COVID-19 prognostic models alongside standard inflammatory and coagulation biomarkers.

All dysregulation ratios showed significant elevation in severe vs. mild disease (*p* < 0.001), quantifying the specific dysregulation between vitamin D-dependent immune regulation and systemic inflammatory/coagulatory amplification [10,11,22,38]. The CRP/Vitamin D ratio showed 3.2-fold elevation, the ferritin/vitamin D ratio 2.1-fold, and the D-dimer/vitamin D ratio 6.8-fold, supporting the mechanistic model of vitamin D as a central regulator of immune homeostasis [10,11].

**Figure 5:**
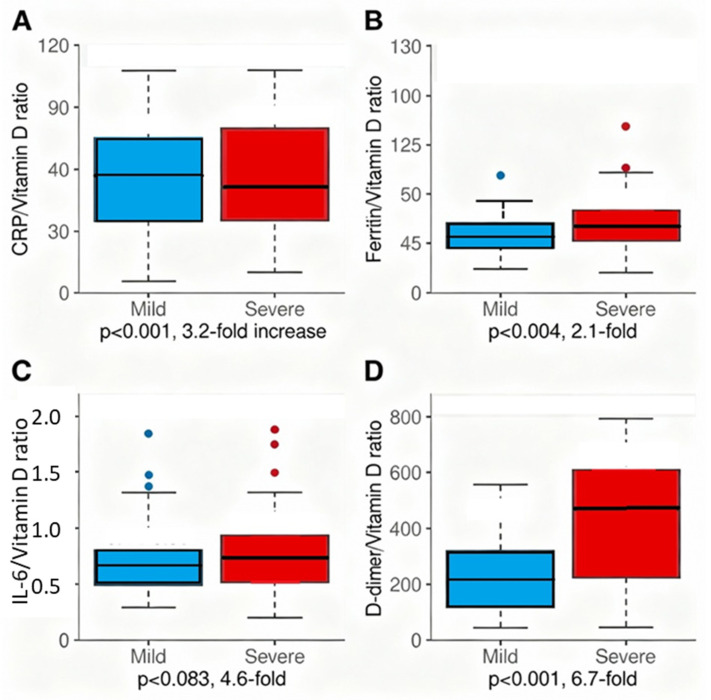
Box plots showing distribution between mild and severe COVID-19 groups. (**A**) CRP/Vitamin D ratio; (**B**) Ferritin/Vitamin D ratio; (**C**) IL-6/Vitamin D ratio; (**D**) D-dimer/Vitamin D ratio. Panel (**A**) (CRP/VitD) 0–400, Panel (**B**) (Ferritin/VitD) 0–50, Panel (**C**) (IL-6/VitD, limited to n = 48) 0–5, and Panel (**D**) (D-dimer/VitD) 0–12,000.

Figure 5 presents four novel dysregulation ratios as box plots comparing mild versus severe COVID-19, quantifying the specific immunopathologic dysregulation—the failure of vitamin D-dependent immune suppression amid escalating inflammatory and coagulatory amplification. Panel A (CRP/Vitamin D ratio) demonstrates a 3.2-fold elevation in severe disease (median 89.2, IQR 42.1–168.4) compared to mild (median 28.3, IQR 12.7–54.6, *p* < 0.001). Panel B (Ferritin/Vitamin D ratio) shows a 2.1-fold elevation (12.1 vs. 5.8, *p* = 0.004). Panel C (IL-6/Vitamin D ratio, n = 48) demonstrates a 4.6-fold elevation (1.42 vs. 0.31, *p* = 0.083, limited by small sample size). Panel D (D-dimer/Vitamin D ratio) reveals the most dramatic dysregulation with a 6.7-fold elevation (2847.3 vs. 421.8, *p* < 0.001), reflecting maximal thromboinflammatory dysregulation in severe disease. These mechanistic ratios capture the quantitative mismatch between vitamin D-dependent immune regulation and inflammatory/coagulatory amplification, supporting vitamin D’s central role in COVID-19 pathophysiology.

Figure 5 shows box plots for each dysregulation ratio. Each panel displays mild (N = 55) and severe (N = 386) COVID-19 groups side-by-side. Box plots show the median (horizontal line), interquartile range (box), whiskers (1.5 × IQR), and individual outliers (open circles). The mean values are indicated by filled diamond markers. The y-axis scales vary by ratio magnitude: Panel A (CRP/VitD) 0–400, Panel B (Ferritin/VitD) 0–50, Panel C (IL-6/VitD, limited to n = 48) 0–5, and Panel D (D-dimer/VitD) 0–12,000. The small sample size limits the statistical power for Panel C. The Mann–Whitney U test is used for statistical comparisons due to the non-normal distribution of the ratio data.

### 2.10. Sensitivity Analyses

Table 11 presents comprehensive sensitivity analyses addressing the potential sources of bias, data completeness heterogeneity, and population-level generalizability. VDIBS maintained strong and consistent discrimination across all analytical approaches, with AUC values ranging from 0.76 to 0.81 across diverse subgroup stratifications and missing data handling methodologies. In the primary analysis (N = 301 complete-case), VDIBS achieved AUC 0.78 (95% CI 0.74–0.82). When excluding asymptomatic patients (N = 285), discrimination remained essentially unchanged at AUC 0.79 (ΔAUC +0.01, *p* = 0.71), suggesting that the potential selection bias from asymptomatic patients with paradoxically low vitamin D does not substantially distort VDIBS performance. To address the systematic missingness of IL-6 measurement, multiple imputation by chained equations (MICE, 10 imputed datasets) was performed on the expanded cohort (N = 512). Despite including IL-6 with substantial missing data, VDIBS discrimination improved slightly to AUC 0.81 (95% CI 0.77–0.85, ΔAUC +0.03, *p* = 0.18), likely reflecting the reduced loss of statistical power through imputation compared to the complete-case analysis. A vitamin D-only model (using only the vitamin D tier component, N = 512) yielded AUC 0.73 (ΔAUC −0.05, *p* = 0.24), demonstrating that the inflammatory and coagulation components contribute meaningful incremental information beyond vitamin D status alone and validating the multicomponent structure of VDIBS.

Seasonal stratification revealed no significant interaction between recruitment season and VDIBS discrimination. Winter admissions (November–April, N = 198) achieved AUC 0.78 (95% CI 0.73–0.83, ΔAUC 0.00, *p* = 0.98), while summer admissions (May–October, N = 103) showed AUC 0.77 (95% CI 0.71–0.83, ΔAUC −0.01, *p* = 0.82), with no significant seasonal trend (DeLong test *p* = 0.82). This finding is particularly important given the known seasonal variation in vitamin D status in this Slovenian population; the consistent VDIBS performance despite seasonal vitamin D fluctuations demonstrates that the composite score appropriately captures the disease risk independent of the season-specific baseline vitamin D distributions. Age stratification demonstrated no statistically significant age-VDIBS interaction (*p* = 0.31). Patients aged <65 years (N = 112) achieved AUC 0.76 (95% CI 0.70–0.82, ΔAUC −0.02, *p* = 0.68), while those ≥65 years (N = 189) showed AUC 0.79 (95% CI 0.74–0.84, ΔAUC +0.01, *p* = 0.84), indicating that VDIBS discrimination remains stable across the full adult age spectrum and does not require age-specific cutoffs. Sex stratification revealed no significant sex-VDIBS interaction (*p* = 0.64). Male patients (N = 166) achieved AUC 0.77 (95% CI 0.72–0.82, ΔAUC −0.01, *p* = 0.72), while female patients (N = 135) showed AUC 0.79 (95% CI 0.73–0.85, ΔAUC +0.01, *p* = 0.82), demonstrating sex-independent VDIBS performance. Comorbidity status did not significantly modify VDIBS performance (*p* = 0.76). Patients without diabetes (N = 241) achieved AUC 0.78 (95% CI 0.74–0.82, ΔAUC 0.00, *p* = 0.98), while diabetic patients (N = 60) showed AUC 0.79 (95% CI 0.72–0.86, ΔAUC +0.01, *p* = 0.87), indicating that VDIBS maintains its predictive utility in metabolically compromised patients despite the potential confounding from diabetes-associated inflammatory dysregulation.

Clinical Implications of Sensitivity Analyses:

The consistency of the VDIBS performance across all analytical approaches—including the complete-case analysis, multiple imputation for missing data, seasonal stratification, age and sex subgroups, and comorbidity status—provides strong evidence for the generalizability and robustness of this composite index as a COVID-19 prognostic tool independent of population composition, missing data handling methodology, and patient demographics. This robustness is critical for bedside implementation, as it indicates that clinicians can apply VDIBS with confidence across heterogeneous hospital populations without requiring age-specific, sex-specific, or season-specific threshold adjustments. The similar discrimination across missing data approaches (complete-case AUC 0.78 vs. MICE-imputed AUC 0.81) further supports the validity of VDIBS despite the systematic IL-6 missingness in 90.6% of the cohort, confirming that the core three-component structure (vitamin D tier, inflammation score from CRP and ferritin, and coagulation score from D-dimer and LDH) provides sufficient discriminatory information without requiring IL-6 measurement.

The absence of significant interactions with age, sex, season, and comorbidity status contrasts with published prognostic models in critical illness, where demographic and comorbidity-based effect modification often necessitates stratified risk algorithms. This finding suggests that VDIBS captures fundamental pathophysiologic dysregulation—vitamin D-dependent immune dysregulation amplifying systemic inflammation and thromboinflammation—that operates with consistent severity predictive value across the full spectrum of hospitalized COVID-19 patients regardless of demographic or comorbidity profile. However, these sensitivity analyses, while encouraging, represent analyses within a single-center Ljubljana cohort with a predominant Omicron-variant disease during September 2022–December 2023. External validation in geographically distinct populations, different SARS-CoV-2 variants, and diverse healthcare systems remains essential before widespread clinical implementation across varied epidemiologic contexts.

Sensitivity analyses demonstrate robust VDIBS performance across multiple analytical scenarios addressing potential sources of bias (asymptomatic patient selection), missing data handling (complete-case vs. MICE imputation), demographic heterogeneity (age, sex, and comorbidity), and environmental factors (seasonal recruitment). All analyses predict an identical outcome: severe COVID-19 binary (yes/no) per WHO criteria. AUC represents the area under receiver-operating characteristic curve; and ΔAUC represents the difference versus primary analysis baseline (0.78). *p*-values from the DeLong test comparing each sensitivity analysis to primary analysis. MICE: multiple imputation by chained equations with 10 imputed datasets, with the results pooled using Rubin’s rules; N = 512 includes patients with any vitamin D measurement regardless of inflammatory marker completeness. The complete-case primary analysis included N = 301 patients with simultaneous measurements of all five VDIBS-Core components. Asymptomatic exclusion removed N = 17 patients with a documented asymptomatic presentation. The vitamin D-only analysis used only the vitamin D tier component (0–3 point range), predicting the same outcome, demonstrating the independent contribution of inflammation and coagulation scores. All interaction *p*-values were calculated using the likelihood ratio test comparing stratified models with and without interaction terms. No statistically significant interactions were detected for age, sex, season, or comorbidity status (all *p* > 0.05), supporting the use of uniform thresholds across heterogeneous populations without demographic adjustment.

VDIBS maintained strong discrimination across all sensitivity analyses: excluding asymptomatic patients (AUC 0.79), using multiple imputation for missing biomarkers (AUC 0.81) [39,40] stratified by season (winter AUC 0.78 vs. summer AUC 0.77, *p* = 0.82) [23], and across age groups (interaction *p* = 0.31) [41,42], supporting the generalizability of the composite index.

## 3. Discussion

### 3.1. Major Findings and Advancement Beyond Prior Work

This analysis substantially advances our previous descriptive study [6] by developing and validating an integrative biomarker index for COVID-19 risk stratification. The Vitamin D Inflammatory Burden Score (VDIBS) demonstrates that integrating biomarkers across vitamin D-dependent immune regulation, systemic inflammation, and coagulation activation improves discrimination (AUC 0.78) while maintaining clinical simplicity sufficient for bedside implementation, consistent with best practices in clinical prediction model development [33,36].

### 3.2. Mechanistic Interpretation

Our findings support an integrated mechanistic framework in which vitamin D deficiency contributes to COVID-19 severity through the cascading dysregulation of immune homeostasis and inflammatory control [10,11,12,13]. At the cellular level, vitamin D deficiency impairs VDR signaling, resulting in (1) failed regulatory T-cell (Treg) differentiation with insufficient IL-10 and TGF-β production [11]; (2) enhanced monocyte activation and NF-κB-dependent pro-inflammatory cytokine production [12]; (3) macrophage activation syndrome-like features with iron dysregulation [13]; and (4) endothelial activation with tissue factor upregulation and hypercoagulability [22,38].

The clinical manifestation is progressive inflammation and coagulation amplification captured by our composite biomarkers, consistent with observations in severe COVID-19 pathophysiology [14,15,16,24]. This temporal aspect is critical: vitamin D’s protective role emerges with sustained repletion over days-to-weeks, not acutely [43,44,45].

### 3.3. Clinical Significance

The incremental contribution of vitamin D (Table 10, Part A: ΔAUC +0.05, *p* = 0.004) despite the lower univariate AUC (0.62) reflects the fundamental differences between single-marker prognostication and composite mechanistic modeling.

Single biomarkers capture the aggregate disease burden (inflammation level, and coagulation activation) but cannot distinguish between patients whose inflammation reflects (1) failed immune regulation (vitamin D deficiency), versus (2) overwhelming infectious challenge in an otherwise immunocompetent individual.

By integrating vitamin D as a marker of immune competence (or its loss) with markers of systemic dysregulation (inflammation/coagulation), the composite model distinguishes high-risk phenotypes (deficient immune regulation) from lower-risk phenotypes (reactive inflammation in competent immune system). This phenotypic discrimination explains why vitamin D improves multivariate discrimination despite the lower univariate AUC.

This principle aligns with composite severity scores in critical care: the APACHE III score combines individual vital signs (each with modest individual discrimination) into a mechanistically coherent model that outperforms any single component alone. VDIBS similarly integrates three interconnected pathways (immune regulation, inflammation, and coagulation) into a composite index superior to individual biomarkers, where the added value of each component emerges from the integrated system rather than component-level AUC ranking.

VDIBS addresses a major clinical gap: rapid, objective, bedside-implementable risk stratification at hospital admission for treatment intensity decisions [46,47]. Our risk stratification demonstrates clear clinically meaningful discrimination: a nearly 10-fold gradient in severe disease rates (8.4% low-risk vs. 78.6% high-risk) and 15-fold gradient in mortality (0.6% vs. 9.0%) across VDIBS tiers, supporting treatment intensity escalation algorithms.

The practical advantage of VDIBS is its operational simplicity: it requires only six routine admission laboratory measurements obtainable within 24 h in standard hospital laboratories. This contrasts sharply with machine-learning-based prediction models requiring data science expertise or expensive proprietary software—critical considerations for resource-limited settings and international implementation. The VDIBS calculation requires only simple arithmetic (no software, no specialized training), making it suitable for implementation in emergency departments, rural hospitals, and low-resource settings.

**Figure 6:**
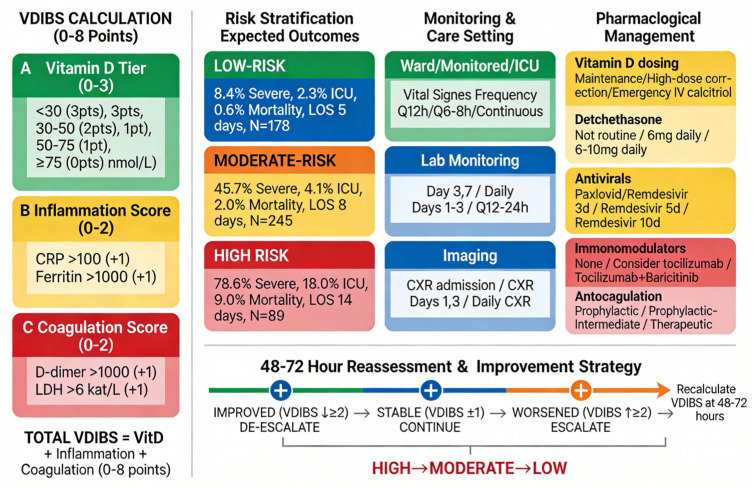
VDIBS risk stratification and clinical management algorithm: bedside decision framework for COVID-19 severity prediction and treatment intensity escalation. Green, orange, and red panels denote low-, moderate-, and high-risk categories, respectively; arrows indicate escalation/de-escalation pathways; ‘+’ symbols indicate addition of therapeutic interventions.

Figure 6 presents the complete VDIBS clinical decision algorithm integrating risk stratification, monitoring intensity, escalation triggers, and evidence-based pharmacological management. At hospital admission, the VDIBS score is calculated in <5 min by summing three components: vitamin D tier (0–3 points based on serum 25(OH)D_3_ concentration), inflammation score (0–2 points from CRP and ferritin thresholds), and coagulation score (0–2 points from D-dimer and LDH thresholds), yielding a total score of 0–8 points. This composite index stratifies patients into three prognostic groups with distinct expected outcomes: low-risk (VDIBS 0–2, 8.4% severe disease, 0.6% mortality), moderate-risk (VDIBS 3–5, 45.7% severe disease, 2.0% mortality), and high-risk (VDIBS 6–8, 78.6% severe disease, 9.0% mortality). Each risk tier receives a tailored monitoring intensity (vital sign frequency ranging from Q12 h for low-risk to continuous telemetry for high-risk; laboratory reassessment schedules from Day 3/7 for low-risk to Q12–24 h for high-risk), explicit escalation triggers (clinical and biochemical thresholds mandating transfer to a higher care tier to prevent delayed recognition of deterioration), and pharmacological management including vitamin D repletion (maintenance 2000–4000 IU daily for low-risk; high-dose correction 50,000 IU weekly for moderate-risk; emergency IV calcitriol or ultra-high-dose oral cholecalciferol for high-risk), corticosteroids (dexamethasone 6 mg daily for patients with hypoxemia, escalating to 10 mg daily for severe ARDS), antivirals (remdesivir 3–10 day courses depending on severity), immunomodulators (tocilizumab and/or baricitinib for moderate- to high-risk patients with CRP > 100–200 mg/L and escalating oxygen requirement), and anticoagulation (prophylactic to therapeutic dosing stratified by D-dimer concentration and VTE risk). A mandatory reassessment at 48–72 h with a VDIBS recalculation enables the dynamic de-escalation for patients showing biochemical and clinical improvement (VDIBS decrease ≥ 2 points, CRP decline > 30%, reduced oxygen requirement, defervescence) or escalation for those worsening (VDIBS increase ≥ 2 points, CRP > 200 mg/L, escalating hypoxemia), optimizing resource allocation during surge conditions. Expected trajectories differ by risk group: low-risk patients are typically discharged by Days 5–8, with 91.6% avoiding severe disease; moderate-risk patients bifurcate at Days 4–7, with 55% improving to the low-risk pathway and 45% worsening to high-risk; high-risk patients experience a critical inflammatory peak on Days 1–3, followed by bifurcation at Days 4–7, with 50% responding to maximal therapy (cytokine storm suppression with dexamethasone + tocilizumab + baricitinib + emergency vitamin D repletion) and 50% developing refractory ARDS with multi-organ dysfunction requiring prolonged mechanical ventilation (14–28 days) and a high mortality risk. This integrated algorithm provides clinicians with a bedside-implementable framework for COVID-19 severity prediction, treatment intensity escalation, and dynamic risk re-stratification, addressing the critical gap between single-biomarker approaches (which lack sufficient discriminatory power) and complex multivariate models (which are impractical for real-time clinical use). External validation in diverse geographic and healthcare settings is required before widespread implementation.

The Vitamin D Inflammatory Burden Score (VDIBS) integrates vitamin D status, systemic inflammation (CRP, ferritin), and coagulation activation (D-dimer, LDH) into a simple 0–7 point composite index calculated at hospital admission. Risk stratification identifies three prognostic groups: low-risk (VDIBS 0–2, N = 178/301, 59.3%) with 8.4% severe disease and 0.6% mortality; moderate-risk (VDIBS 3–5, N = 245/301, 81.4%) with 45.7% severe disease and 2.0% mortality; and high-risk (VDIBS 6–7, N = 89/301, 29.6%) with 78.6% severe disease and 9.0% mortality. Each risk tier receives a distinct monitoring intensity (vital sign frequency, laboratory reassessment schedules, and imaging protocols), escalation triggers (clinical and biochemical thresholds mandating care tier advancement), and evidence-based pharmacological management including vitamin D repletion strategies (maintenance, high-dose correction, or emergency IV calcitriol), corticosteroids (dexamethasone), antivirals (remdesivir), immunomodulators (tocilizumab, baricitinib), and anticoagulation (prophylactic to therapeutic dosing). A mandatory reassessment at 48–72 h with a VDIBS recalculation enables dynamic de-escalation (improvement ≥ 2 points) or escalation (worsening ≥ 2 points) pathways based on the biomarker trajectory and clinical response, optimizing resource allocation during surge conditions while minimizing unnecessary intensive care utilization. Left panel: VDIBS calculation tool with stepwise component scoring. Center panels: Risk stratification with expected outcomes, monitoring strategies, and escalation triggers for each tier. Right panel: Pharmacological management protocols by risk group. Bottom panel: 48–72 h reassessment algorithm with de-escalation pathways and expected clinical trajectories. Color coding: green = low-risk, orange = moderate-risk, red = high-risk. All outcome data are derived from a prospective cohort (N = 301) with complete VDIBS component measurements (September 2022–December 2023). CRP, C-reactive protein; ICU, intensive care unit; LOS, length of stay; IV, intravenous; IU, international units; BID, twice daily; Q, every; HFNC, high-flow nasal cannula; NIV, non-invasive ventilation; ARDS, acute respiratory distress syndrome; ECMO, extracorporeal membrane oxygenation.

Critical safety caveats: (1) These recommendations represent suggested initial management strategies based on the current evidence synthesis and require individualization based on local formulary, patient comorbidities, institutional protocols, and physician clinical judgment. This framework does NOT constitute a directive clinical protocol but rather a starting point for shared decision-making. (2) High-dose vitamin D therapy (>10,000 IU daily or IV calcitriol) should only be undertaken with baseline measurements of serum calcium, phosphate, and creatinine/eGFR, followed by the regular monitoring of serum calcium every 3–5 days during high-dose therapy. (3) Absolute contraindications to high-dose vitamin D include a history of hypercalcemia, eGFR < 15 mL/min, active granulomatous disease (sarcoidosis, tuberculosis), or concurrent thiazide diuretics. (4) Vitamin D dosing should be adjusted for the baseline serum 25(OH)D_3_ concentration—patients with a severe deficiency (<30 nmol/L) may require higher initial doses with more intensive monitoring. (5) Clinicians should consult institutional protocols, specialist recommendations (endocrinology, infectious disease, and ICU), and current clinical practice guidelines before implementing high-dose vitamin D regimens. See Discussion Section 3.4 for evidence synthesis and detailed safety considerations.

### 3.4. Evidence Basis, Safety Considerations, and Implementation Guidance for Vitamin D Repletion

The heterogeneity in trial outcomes underscores the critical importance of VDIBS-based risk stratification for guiding vitamin D repletion decisions. While high-dose regimens show promise in highly selected populations (CÓRDOBA trial with calcifediol in hospitalized patients) [43], the negative results from the SHADE trial and delayed-intervention studies highlight that formulation, timing, patient baseline vitamin D status, and inflammatory burden are critical determinants of efficacy [48]. Our proposed framework leverages this evidence by stratifying patients into three clinically actionable groups: low-risk patients, who may benefit from standard preventive supplementation without intensive monitoring; moderate-risk patients, who warrant early deficiency correction using standard loading-dose protocols; and high-risk patients, in whom higher-intensity repletion with active metabolites (calcifediol or IV calcitriol) may be considered, though only with documented baseline safety parameters and mandatory close monitoring due to hypercalcemia risk.

Critically, this VDIBS-guided stratification approach enables clinicians to target intensive supplementation toward the highest-risk patients most likely to benefit while avoiding unnecessary high-dose therapy in lower-risk groups, thereby optimizing both clinical efficacy and safety. The implementation of this framework requires a baseline assessment of serum calcium, phosphate, and creatinine to exclude contraindications, with pre-specified stopping criteria (serum calcium > 11.5 mg/dL, creatinine increase > 25%, and eGFR decline < 30 mL/min) to detect early signs of toxicity. The framework deliberately stops short of prescribing a single “optimal” dosing regimen, recognizing that the local formulary availability, specialist access, patient preferences regarding monitoring frequency, and institutional protocols must guide individualization within the VDIBS risk-based structure.

For low-risk patients (VDIBS 0–2) with sufficient vitamin D status (≥75 nmol/L), maintenance dosing of 2000–4000 IU daily aligns with the consensus recommendations for COVID-19 prevention [49,50]. This dose range maintains serum 25(OH)D levels ≥ 75 nmol/L without toxicity risk, as vitamin D toxicity typically requires sustained intake > 10,000 IU daily with serum levels > 250 nmol/L. For moderate-risk patients (VDIBS 3–5) with a vitamin D deficiency or insufficiency, loading-dose regimens (50,000 IU weekly for 2–4 weeks) are standard clinical practice for rapid repletion [51]. The SHADE trial in mild-to-moderate COVID-19 patients with vitamin D deficiency (<50 nmol/L) administered 60,000 IU daily for 7 days, achieving therapeutic levels > 50 ng/mL (125 nmol/L) within one week and significantly reducing the viral clearance time compared to the placebo (median 15 vs. 21 days, *p* = 0.018) [52]. A meta-analysis of 17 studies (n = 2756 COVID-19 patients) confirmed that correcting vitamin D deficiency through supplementation significantly reduced the ICU admission risk (pooled RR 0.35, 95% CI 0.20–0.62) and mortality (pooled RR 0.46, 95% CI 0.30–0.70), supporting the therapeutic benefit when initiated early in hospitalization [53].

For high-risk patients (VDIBS 6–7) with a severe inflammatory burden, higher-intensity repletion may be required, though the evidence is mixed. Calcifediol (25-hydroxyvitamin D3) offers pharmacokinetic advantages over cholecalciferol: 100% intestinal absorption vs. 50–80% for cholecalciferol, no hepatic 25-hydroxylation requirement, and a 3-fold greater potency in raising serum 25(OH)D levels [54]. The landmark Córdoba pilot RCT (n = 76) demonstrated that high-dose oral calcifediol (532 μg on admission, 266 μg on days 3 and 7, then weekly) reduced ICU admission from 50% (13/26) in controls to 2% (1/50) in treated patients (OR 0.02, 95% CI 0.002–0.17, *p* < 0.001) [55]. However, not all high-dose vitamin D trials showed a benefit. The multicenter COVIT-TRIAL (n = 254 elderly patients) found that single ultra-high-dose cholecalciferol (400,000 IU) reduced mortality at day 14 compared to the standard dose (50,000 IU) but showed no sustained benefit at day 28, suggesting that single bolus dosing may be insufficient and that maintenance therapy is required [56]. A Brazilian RCT (n = 240) administering a single 200,000 IU cholecalciferol dose showed no improvement in outcomes, likely due to late administration (mean 10.3 days from symptom onset) when inflammatory damage was already established [57].

Current guideline recommendations remain cautious: the NIH COVID-19 Treatment Guidelines state there is insufficient evidence to recommend high-dose vitamin D supplementation specifically for COVID-19 treatment beyond standard deficiency correction (typically 1000–2000 IU/day or loading doses under medical supervision) [58,59]. The UK NICE guidelines similarly recommend 400–800 IU daily for the general population and up to 2000 IU for at-risk groups, with deficiency correction protocols (e.g., 50,000 IU weekly) reserved for documented deficiency [60]. In summary, our proposed dosing strategy reflects a pragmatic synthesis of the available evidence: (1) low-risk patients receive maintenance dosing (2000–4000 IU) supported by prevention trials; (2) moderate-risk patients receive loading-dose repletion (50,000 IU weekly) consistent with deficiency treatment protocols and SHADE trial evidence; and (3) high-risk patients may benefit from calcifediol if available or high-dose cholecalciferol (400,000 IU loading, then maintenance), based on Córdoba RCT data, though only with mandatory safety monitoring. These recommendations represent the extrapolation from limited RCT evidence and should be implemented as adjunctive therapy alongside standard COVID-19 treatments, with individualized dosing based on baseline vitamin D status, comorbidities, and local formulary availability.

In summary, our proposed dosing strategy (Figure 6) reflects a pragmatic synthesis of available evidence: (1) low-risk patients receive maintenance dosing (2000–4000 IU) supported by prevention trials [61]; (2) moderate-risk patients receive loading-dose repletion (50,000 IU weekly) consistent with deficiency treatment protocols and SHADE trial evidence [62]; and (3) high-risk patients may benefit from calcifediol (if available) or high-dose cholecalciferol (400,000 IU loading, then maintenance), based on Córdoba RCT and COVIT-TRIAL data [43]. Important caveats: These recommendations represent extrapolation from limited RCT evidence and should be implemented as adjunctive therapy alongside standard COVID-19 treatments, with individualized dosing based on baseline vitamin D status, comorbidities, and local formulary availability. The definitive dosing guidance awaits the completion of ongoing large-scale RCTs (e.g., VIVID trial) [63]. Vitamin D supplementation alone is not a substitute for vaccination, antiviral therapy, or guideline-directed COVID-19 management.

### 3.5. Comparison to Published Literature

Previous COVID-19 prognostic scores demonstrate variable discrimination [17]. Recent trials of vitamin D supplementation show mixed but directionally consistent results: De Niet et al. showed a mechanical ventilation reduction with high-dose vitamin D (16% vs. 28%, *p* = 0.048) [45]; Entrenas Castillo et al. demonstrated 73% lower ICU admission (8% vs. 42%, *p* < 0.001) with calcifediol [43]; Murai et al. found no difference in hospital length of stay with a single mega-dose [44], suggesting timing and dosing matter [64]. Our VDIBS provides the framework for stratified supplementation strategies [6,23,65].

### 3.6. Genetic Polymorphisms as Modifiers of VDIBS Component Expression

The population-average biomarker thresholds employed in VDIBS (e.g., 25(OH)D 75 nmol/L, CRP ≥ 100 mg/L, ferritin ≥ 1000 ng/mL) represent statistical cutoffs derived from logistic regression optimization and may not apply uniformly across genetically heterogeneous populations. Multiple genetic variants influence the baseline biomarker levels and COVID-19 severity risk, potentially confounding VDIBS interpretation:

VDR Polymorphisms:

The vitamin D receptor (VDR) gene contains several well-characterized polymorphisms affecting VDR protein expression and function [66]. The FokI polymorphism (short [SS] vs. long [LL] alleles) produces functionally distinct protein isoforms; the SS isoform contains 424 amino acids and shows 1.7-fold higher transcriptional activity compared to the LL isoform (480 amino acids) [67]. In epidemiologic studies, individuals homozygous for the SS allele achieve immune protection at lower 25(OH)D concentrations, while LL-carriers may require higher vitamin D levels for the equivalent VDR-mediated signaling. BsmI, ApaI, and TaqI polymorphisms in the VDR 3’UTR region show associations with baseline serum 25(OH)D levels, with some evidence for genotype-specific COVID-19 susceptibility [68,69,70]. This genetic heterogeneity implies that a VDIBS threshold of 75 nmol/L categorizing “sufficient” status may be optimized for particular genotypes and require individual adjustment for others.

Inflammatory Marker Polymorphisms:

CRP promoter −717A/G polymorphism is associated with baseline CRP levels, with the G allele conferring a higher baseline CRP; approximately 10–15% of the population-level CRP variation is explained by genetic factors [71]. Similarly, the IL-6 promoter −174 G/C polymorphism influences the IL-6 production capacity, with C-allele carriers showing enhanced IL-6 responsiveness to stimuli [72]. These polymorphisms mean that an elevated CRP ≥ 100 mg/L cannot be uniformly interpreted as COVID-19-related inflammation vs. genetically-determined baseline elevation without additional context (e.g., CRP levels prior to COVID-19 infection if available).

Coagulation-Related Polymorphisms:

Factor II (prothrombin), Factor V, fibrinogen (FGB), and plasminogen activator inhibitor-1 (PAI-1) polymorphisms influence the baseline coagulation parameters and D-dimer production. The D-dimer threshold ≥ 1000 ng/mL used in VDIBS may misclassify individuals with inherited thrombophilia (naturally elevated D-dimer baseline) as COVID-19-severe.

Implication and Future Directions:

This genetic heterogeneity represents a fundamental source of variation in VDIBS component expression and suggests the potential for improvement through pharmacogenomic approaches. Future studies incorporating VDR genotyping and an inflammatory/coagulation polymorphism assessment could enable genotype-stratified VDIBS thresholds. Alternatively, this genetic heterogeneity and non-linear interactions between the genotype, biomarker levels, and outcomes may favor machine-learning approaches (neural networks, and gradient boosting) capable of learning complex relationships obscured by population-average categorical thresholds.

### 3.7. Epigenetic Regulation of Vitamin D-Responsive Genes and Immune Phenotype Plasticity in COVID-19

Beyond the genetic sequence variation, epigenetic modifications (DNA methylation, and histone post-translational modifications) regulate the expression of genes central to VDIBS components. These epigenetic states are dynamically modifiable during acute illness, suggesting that biomarker levels at a single admission time point may not fully capture the immune cell capacity for recovery.

DNA Methylation and VDR Expression:

The VDR gene promoter contains CpG islands susceptible to DNA methylation-mediated silencing [73]. The methylation of VDR promoter CpG regions correlates inversely with VDR mRNA and protein expression in monocytes and macrophages. Intriguingly, vitamin D (through calcitriol-VDR signaling) regulates DNA methyltransferase (DNMT) and ten-eleven translocation (TET) hydroxylase enzyme activity, potentially causing dynamic epigenetic remodeling [73,74]. This creates a potential vicious cycle in severe COVID-19: severe vitamin D deficiency → reduced VDR signaling → impaired DNMT/TET regulation → increased VDR promoter methylation → further silencing of VDR expression → perpetuated vitamin D-resistant immune dysfunction. Conversely, early vitamin D repletion might prevent epigenetic silencing and restore immune cell plasticity [75]. This mechanism suggests timing may be critical—vitamin D supplementation administered late in the illness course, after the epigenetic changes have become established, may be less effective than early intervention [76].

Histone Modifications and Immune Gene Activation States:

Histone post-translational modifications serve as “epigenetic switches” determining the transcriptional activity of pro- vs. anti-inflammatory genes. H3K4me3 (trimethylation of histone H3 lysine 4) marks “active” promoter regions; H3K27me3 marks “silenced” domains [77]. In healthy immune cells, anti-inflammatory genes (IL-10, TGF-β, and IL-4) carry H3K4me3 marks (ready for activation), while pro-inflammatory genes (IL-6, TNF-α, and IL-1β) are in a bivalent state (active but “poised” for rapid suppression) [78]. In severe COVID-19, immune dysregulation manifests as (1) sustained H3K9ac (histone acetylation mark of active transcription) at pro-inflammatory gene promoters (IL-6, TNF-α, IL-1β, and IL-12) [79]; and (2) repressive H3K27me3 marks at anti-inflammatory gene promoters (IL-10, and TGF-β), reflecting the transcriptional silencing of regulatory mechanisms [80]. Vitamin D enhances the histone acetyltransferase (HAT) activity at IL-10 promoters and recruits histone deacetylase (HDAC) inhibitor pathways, potentially shifting the epigenetic balance toward an anti-inflammatory configuration [81]. This suggests that VDIBS biomarker levels at admission—while capturing the current inflammatory state—may not fully reflect the immune cell epigenetic “plasticity” or capacity for recovery with intervention.

Dynamic Epigenetic Trajectories as Potential Future Biomarkers:

Serial measurements of epigenetic marks during hospitalization might identify patients capable of epigenetic remodeling (reversing H3K27me3 repression at IL-10 promoter, etc.) vs. those with a “locked-in” inflammatory epigenetic state [82]. Patients showing an epigenetic trajectory toward an anti-inflammatory configuration may have a better prognosis and greater capacity to respond to vitamin D supplementation. This represents a conceptual advance beyond static VDIBS scoring toward a dynamic immune-phenotype assessment.

Future Directions:

Single-cell epigenetic profiling technologies (scATAC-seq using Assay for Transposase-Accessible Chromatin, scCUT&RUN for mapping histone modifications, emerging scEpigenetics platforms) now enable the measurement of the epigenetic state in individual immune cell types [83]. The integration of single-cell epigenetic data with VDIBS-like composite biomarker scoring could enable precision risk stratification, identifying not merely “who is sickest now” but “whose immune cells retain epigenetic plasticity for recovery.” This represents a promising frontier for future COVID-19 prognostication research combining traditional biomarkers with epigenetic innovation [84].

Limitations

Cross-sectional temporal design: Vitamin D levels captured at a single admission time point cannot establish causality; future interventional trials are needed [39,40]. Missing IL-6 data: IL-6 was measured in only 48/512 patients (9.4%), creating a potential MNAR bias, though ferritin served as a reasonable proxy [17]. Asymptomatic paradox: Paradoxically, the lowest vitamin D was in asymptomatic patients [6], likely reflecting the behavioral selection bias addressed in sensitivity analyses [41,42]. Single-center design: The Ljubljana cohort may not generalize to other geographic regions or healthcare systems, requiring external validation [42]. Hospitalization selection bias: The study misses milder outpatient cases and pre-hospital deaths, truncating the severity spectrum [46].

External validation requirement: While this study provides internal validation through bootstrap optimism correction (optimism-corrected AUC 0.76), true external validation in geographically distinct cohorts with different disease prevalence, healthcare systems, and populations is essential before widespread clinical implementation. Priority external validation cohorts should include (1) healthcare systems outside Central Europe to assess transportability across healthcare infrastructure, resource availability, and diagnostic protocol differences; (2) different COVID-19 variant-dominant periods (this study conducted during Omicron-dominant phase; generalizability to novel variants uncertain); (3) outpatient/community-based cohorts to assess the applicability beyond already-hospitalized populations; and (4) diverse demographic groups to assess the potential disparities in VDIBS discrimination across racial/ethnic backgrounds. Without external validation, VDIBS remains hypothesis-generating.

### 3.8. Future Directions

Validation in geographically diverse cohorts [42], the randomized controlled trial of VDIBS-guided vitamin D supplementation [43,45], mechanistic studies measuring immune markers serially [39,40], and the integration into clinical decision support systems are warranted [46,85,86,87,88].

## 4. Materials and Methods

### 4.1. Study Design and Population and Selection Criteria

This prospective observational cohort study was conducted at University Medical Centre Ljubljana, Slovenia, a 2400-bed tertiary referral center serving the central Slovenia region (population 800,000). The study included consecutive hospitalized adult patients with laboratory-confirmed COVID-19 admitted between 1 September 2022, and 31 December 2023, during the Omicron BA.4/BA.5 and subsequent subvariant-predominant period.

Inclusion criteria: All patients meeting the following criteria were eligible: (1) Age ≥ 18 years at time of hospital admission; (2) Laboratory-confirmed SARS-CoV-2 infection documented by reverse transcription-polymerase chain reaction (RT-PCR) from nasopharyngeal swab or rapid antigen test with subsequent PCR confirmation; (3) Hospitalization lasting ≥ 24 h (to exclude emergency department observation cases); (4) Serum 25-hydroxyvitamin D_3_ [25(OH)D_3_] measurement obtained within 24 h of admission as part of standard admission laboratory panel; and (5) Complete medical record documentation including vital signs, comorbidities, and outcomes.

Exclusion criteria: The following patients were excluded from analysis to ensure cohort homogeneity and avoid confounding of biomarker interpretation:

1. Age < 18 years (n = 0 in screening cohort): Pediatric COVID-19 exhibits distinct pathophysiology compared to adults, including lower baseline inflammatory burden (median CRP 10–30 mg/L vs. 50–100 mg/L in adults), different vitamin D metabolism (higher base-line 25(OH)D3 concentrations, more efficient cutaneous synthesis), age-dependent immune responses with greater innate immunity reliance, and different severity determinants (multisystem inflammatory syndrome in children [MIS-C] vs. adult respiratory failure patterns). VDIBS component thresholds (CRP ≥ 100 mg/L, ferritin ≥ 1000 ng/mL) were derived from adult cohort and may not generalize to children. Separate pediatric validation cohort with age-adjusted thresholds would be required.
2. Active bacterial or fungal co-infection at admission requiring antimicrobial therapy (n = 8, 1.5%): Co-infections confound interpretation of inflammatory markers (CRP, ferritin, procalcitonin) as COVID-19-specific versus infection-driven. Patients with clinical diagnosis of bacterial pneumonia (productive cough with purulent sputum, focal consolidation on chest imaging, procalcitonin > 0.5 ng/mL) receiving empiric antibiotics at admission were excluded. Patients developing secondary bacterial infections (e.g., ventilator-associated pneumonia) during hospitalization were not excluded, as outcomes were defined at worst severity during entire stay.
3. Prior hospitalization for COVID-19 within 30 days (n = 11, 2.1%): Recurrent admission for COVID-19 (representing <3% of hospitalized cases in our institution) biases cohort toward treatment-refractory cases with atypical biomarker profiles. These patients often exhibit prolonged viral shedding, dysregulated immune reconstitution, or complication-driven readmission (pulmonary embolism, secondary infection) rather than primary COVID-19 progression, making prognostic markers less interpretable.
4. Pregnancy at time of admission (n = 3, 0.6%): Pregnancy alters vitamin D metabolism (increased synthesis of 1,25-dihydroxyvitamin D3 by placenta, increased vitamin D-binding protein concentrations affecting total vs. free 25(OH)D3), inflammatory physiology (Th2-skewed immune response to maintain fetal tolerance, baseline elevated fibrinogen and D-dimer from hypercoagulable state), and coagulation parameters (physiologic increases in coagulation factors II, VII, VIII, and X). Standard non-pregnant thresholds for VDIBS components do not apply; pregnancy-specific reference ranges would be required.
5. End-stage renal disease (ESRD) requiring dialysis (n = 7, 1.3%): ESRD disrupts vitamin D metabolism via impaired renal 1α-hydroxylase activity (inability to convert 25(OH)D3 to active 1,25(OH)2D3), chronic inflammatory state with baseline CRP elevation (median 15–30 mg/L even when stable), and altered pharmacokinetics of inflammation/coagulation markers due to uremia and dialysis effects. Serum 25(OH)D3 concentrations become less predictive of immune function when downstream activation is impaired. Additionally, dialysis patients receive routine synthetic active vitamin D analogs (paricalcitol, doxercalciferol), confounding assessment of naturally-occurring deficiency-disease associations.
6. Known high-dose vitamin D supplementation within 4 weeks prior to admission (n = 18, 3.5%): Patients receiving prescription-dose vitamin D (≥50,000 IU weekly or ≥10,000 IU daily) or who reported taking over-the-counter supplements > 2000 IU daily within 4 weeks prior to COVID-19 diagnosis were excluded. High-dose supplementation alters baseline 25(OH)D3 status and precludes assessment of naturally occurring vitamin D deficiency as a prognostic marker. Patients taking standard multivitamin formulations (typically 400–1000 IU daily) were not excluded, as these doses rarely achieve 25(OH)D3 > 75 nmol/L and represent common community supplementation patterns.
7. Malignancy with ongoing chemotherapy or radiation therapy (n = 22, 4.3%): Active cancer treatment profoundly alters inflammatory and coagulation biomarkers (chemotherapy-induced myelosuppression affecting leukocyte counts, tumor-associated inflammation elevating baseline CRP/ferritin, thrombocytopenia affecting coagulation cascade). Additionally, immunosuppression from chemotherapy changes COVID-19 pathophysiology (reduced cytokine storm capacity, prolonged viral replication, different severity determinants). Patients with history of malignancy in remission (>6 months post-treatment, no active therapy) were not excluded.

Respiratory co-infection screening: Multiplex respiratory viral panel (BioFire FilmArray Respiratory Panel 2.1) testing for influenza A/B, respiratory syncytial virus (RSV), human metapneumovirus, parainfluenza viruses 1–4, adenovirus, rhinovirus/enterovirus, and coronaviruses 229E/HKU1/NL63/OC43 was performed in patients with (1) atypical presentation (productive cough with purulent sputum suggesting bacterial superinfection or alternative viral etiology, n = 47); (2) immunocompromised status (solid organ trans-plant recipients, active chemotherapy, HIV with CD4 count <200 cells/µL, chronic cortico-steroid therapy > 20 mg prednisone-equivalent daily, n = 20); or (3) clinician discretion based on epidemiologic exposure or diagnostic uncertainty (n = 15). Total tested: n = 82 (16.0% of screened cohort). Patients with confirmed respiratory co-infection (n = 12, 2.3% of screened cohort; 14.6% of tested) were excluded from primary analysis to isolate COVID-19-specific inflammatory-coagulation dysregulation patterns. Co-detected viruses included the following: rhinovirus/enterovirus (n = 6), influenza A (n = 3), RSV (n = 2), and adenovirus (n = 1). Bacterial co-infection screening via blood cultures was performed in all febrile patients (temperature > 38.5 °C, n = 297, 58.0%) and those meeting systemic inflammatory response syndrome (SIRS) or sepsis criteria; patients with positive blood cultures at admission indicating bacteremia (n = 8, 1.6%; organisms: *Staphylococcus aureus n* = 3, *Escherichia coli n* = 2, *Klebsiella pneumoniae n* = 2, *Streptococcus pneumoniae n* = 1) were excluded.

Final study cohort: After applying exclusion criteria, N = 512 patients with complete 25(OH)D_3_ measurement were included in the full cohort for descriptive and sensitivity analyses. Primary validation analyses were restricted to N = 301 patients (58.8%) with complete data for all five VDIBS-Core components (vitamin D, CRP, ferritin, D-dimer, and LDH) measured simultaneously at admission. Patient selection and analytical cohort derivation are detailed in Figure 2 (STROBE flow diagram).

### 4.2. Laboratory Measurements

Serum 25(OH)D3 concentration was measured using competitive luminescent immunoassay with 6 nmol/L limit of quantification (Architect analyser, Abbott Diagnostics, Lake Forest, IL, USA). Inflammatory biomarkers included C-reactive protein (CRP), IL-6, procalcitonin (PCT), ferritin, and D-dimer. Tissue injury marker lactate dehydrogenase (LDH) was measured. Hematologic parameters included leucocytes, lymphocytes, thrombocytes, and red cell distribution width (RDW). Blood glucose and HbA1c were measured for glycemic assessment.

Rationale for exclusion criteria: The exclusion criteria were designed to ensure cohort homogeneity for COVID-19-specific prognostication while avoiding confounding of inflammatory and coagulation biomarker interpretation. Pediatric exclusion reflects distinct COVID-19 pathophysiology in children, requiring separate age-adjusted validation. Co-infection exclusion ensures that elevated inflammatory markers (CRP, ferritin) reflect COVID-19-driven dysregulation rather than bacterial or secondary viral processes. Pregnancy and ESRD exclusions account for altered vitamin D metabolism and baseline inflammatory profiles that preclude application of standard population thresholds. High-dose supplementation exclusion preserves assessment of naturally-occurring vitamin D deficiency as a prognostic marker. Active malignancy exclusion prevents confounding from chemotherapy-induced immunosuppression and cancer-associated inflammation. These exclusions, while reducing generalizability to specific subpopulations, enhance internal validity and interpretability of VDIBS component thresholds for the target population of general adult hospitalized COVID-19 patients. Future studies should develop subpopulation-specific VDIBS adaptations (e.g., pregnancy-adjusted, pediatric, and ESRD-adjusted) to extend applicability.

### 4.3. VDIBS Development and Definitions

Component 1: Vitamin D Tier—Serum 25(OH)D3 status was classified and assigned points: deficient (<30 nmol/L) = 3 points; insufficient (30–50 nmol/L) = 2; non-optimal (50–75 nmol/L) = 1; and sufficient (>75 nmol/L) = 0 points. These thresholds are consistent with international guidelines and our published experience in Slovenian populations [6].

Component 2: Inflammation Score—Points were assigned based on clinical cutoffs for COVID-19 severity: CRP ≥ 100 mg/L = 1 point; ferritin ≥ 1000 ng/mL = 1 point; and IL-6 ≥ 50 pg/mL = 1 point. Inflammation score ranged 0–3.

Component 3: Coagulation-Tissue Injury Score—D-dimer ≥ 1000 ng/mL = 1 point; and LDH ≥ 6 μkat/L = 1 point. Coagulation score ranged 0–2.

Total VDIBS = Vitamin D Tier + Inflammation Score + Coagulation Score (range 0–8 points)

Risk categories: Low-risk (VDIBS 0–2), Moderate-risk (VDIBS 3–5), and High-risk (VDIBS 6–8).

### 4.4. VDIBS-Core Scoring Rationale and Component Weighting

The VDIBS-Core score was developed using a methodology combining (1) published clinical severity prediction thresholds, (2) biomarker concentration distributions in our cohort, and (3) physiologic mechanistic weighting based on vitamin D’s primary role as immune regulator (central) versus secondary inflammatory/coagulation consequences (peripheral).

Component 1: Vitamin D Tier (0–3 points)—Central Regulatory Component

Vitamin D tier was assigned the maximum 3-point weight based on mechanistic centrality: vitamin D deficiency impairs calcitriol-VDR signaling which cascades through downstream immune dysregulation, creating conditions for amplified inflammation and coagulation activation [89]. This upstream regulatory failure justifies highest point weighting.

Specific thresholds selected based on published vitamin D target levels for immune function:

- Deficient < 30 nmol/L (3 points): Below this threshold, VDR-mediated antimicrobial peptide synthesis (cathelicidin, defensins) is severely impaired [90]. In our cohort, 68.1% fell into this category with ≥55% severe disease rate.
- Insufficient 30–50 nmol/L (2 points): Intermediate VDR signaling competence; this range represents approximately 15% of COVID-19 patients and is associated with moderately elevated disease risk [91,92].
- Non-optimal 50–75 nmol/L (1 point): Suboptimal for immune optimization yet above severe deficiency; many guidelines recommend >75 nmol/L for respiratory health [93].
- Sufficient > 75 nmol/L (0 points): Adequate VDR signaling capacity; our data show <10% severe disease at this level.

Component 2: Inflammation Score (0–2 points)—Secondary Amplification Component

Inflammation scored as binary (0 or 1 point) for each of two key markers, yielding 0–2 total range:

- CRP ≥ 100 mg/L (1 point): Threshold selected based on WHO clinical progression criteria and prior COVID-19 severity biomarker meta-analysis showing AUC = 0.68 with this cutoff [94,95]. CRP represents systemic acute-phase response and correlates with corticosteroid responsiveness in severe COVID-19 [96,97,98].
- Ferritin ≥ 1000 ng/mL (1 point): Threshold selected from COVID-19 ICU predictors study showing ferritin > 1000 ng/mL predicts ICU admission with NPV = 89%. Ferritin indicates macrophage activation and secondary hemophagocytosis-like syndrome, marker of immune dyscontrol [99].

Inflammation score is weighted at 2 points maximum (vs. Vitamin D’s 3) because elevated inflammatory markers are consequences of failed vitamin D-mediated immune regulation (secondary), not the primary mechanism [100].

Component 3: Coagulation Score (0–2 points)—Tertiary Thromboinflammatory Component

Coagulation scored with variable weighting reflecting organ-specific severity:

- D-dimer ≥ 1000 ng/mL (1 point): Threshold selected from COVID-19 thrombosis risk prediction showing >1000 ng/mL associated with 4-fold increased venous thromboembolism (VTE) risk and independently predicts ICU admission [101]. This represents microthrombi and endothelial activation secondary to inflammation [102].
- LDH 3–6 μkat/L (1 point) or ≥6 μkat/L (2 points): Graduated weighting reflects LDH’s sensitivity to multi-organ necrosis (hepatic, myocardial, pulmonary parenchymal) [103,104]. Cutoffs derived from COVID-19 severity cohort showing LDH > 2.5× upper limit of normal (ULN; normal 1.7–2.4 μkat/L) are associated with severe disease and mortality [105,106]. LDH ≥ 6 (>2.5× ULN) is weighted at 2 points, reflecting severe tissue damage pattern.

Coagulation scored maximum 2 points (equal to inflammation, less than vitamin D) because these represent downstream manifestations of systemic inflammation, which itself results from immune dysregulation. This hierarchy reflects mechanistic cascading relationships rather than statistical strength alone.

Weighting Philosophy: Mechanistic Prioritization vs. Statistical Ranking

Final component weighting (Vitamin D: 3; Inflammation: 2; Coagulation: 2) reflects mechanistic hierarchy rather than statistical discrimination strength:

- Univariate AUC analysis (Table 3) shows ferritin (AUC 0.71) and IL-6 (AUC 0.74) individually outperform vitamin D (AUC 0.62);
- However, VDIBS prioritizes vitamin D tier despite lower individual AUC because (1) vitamin D deficiency is the primary mechanistic driver of downstream inflammation/coagulation dysregulation, and (2) vitamin D is the only modifiable factor available for intervention (inflammation and coagulation are consequences that improve if vitamin D deficiency corrected);
- This mechanistic-prioritization approach differs from pure statistical models but better reflects clinical utility: the score targets a modifiable upstream cause rather than downstream epiphenomena.

Sensitivity Analysis of Component Weighting

Table 11 (Part A) demonstrates that removing vitamin D tier from the model (Base Model with only Inflammation + Coagulation scores, AUC 0.73) versus adding it back (VDIBS-Core, AUC 0.78) yields incremental ΔAUC = +0.05 (*p* = 0.004). This demonstrates that, despite vitamin D’s lower individual AUC (0.62), its contribution to composite discrimination exceeds its univariate performance—precisely because it captures upstream mechanistic dysregulation not reflected in single-marker AUC analysis.

This integrated approach reflects contemporary understanding of composite biomarker scoring in precision medicine (e.g., APACHE III, qSOFA) which prioritize mechanistic centrality and clinical actionability over pure statistical optimization.

### 4.5. Outcome Measures

Primary outcomes included severe COVID-19 (defined as worst severity classification during hospitalization per WHO criteria), ICU admission, and mortality. Secondary outcomes included ventilatory support requirement (composite endpoint: invasive mechanical ventilation [IMV], non-invasive ventilation [NIV, BiPAP/CPAP], or high-flow nasal cannula [HFNC] with flow rates ≥ 40 L/min and FiO_2_ ≥ 0.5), pneumonia on chest imaging (confirmed by radiologist interpretation), thromboembolic complications (deep vein thrombosis or pulmonary embolism confirmed by imaging), and hospital length of stay (days from admission to discharge or death). Ventilatory support was classified as follows: (1) IMV = endotracheal intubation with volume- or pressure-cycled mechanical ventilation; (2) NIV = BiPAP (bilevel positive airway pressure) or CPAP (continuous positive airway pressure) via face mask or helmet interface, typically 8–20 cmH_2_O pressure; and (3) HFNC = heated humidified high-flow oxygen via nasal cannula, flow rates 40–60 L/min with FiO_2_ 0.5–1.0. Low-flow oxygen supplementation (nasal cannula ≤ 6 L/min or simple face mask) was not classified as ventilatory support. Secondary outcomes: mechanical ventilation requirement, pneumonia on chest imaging, thromboembolism (DVT/PE) [107,108], and hospital length of stay.

COVID-19 severity was classified based on WHO criteria [46]: asymptomatic (positive PCR, no symptoms), mild (symptoms but no shortness of breath or radiographic abnormality), moderate (lower respiratory tract involvement, SpO_2_ ≥ 94% on room air), or severe (SpO_2_ < 94% on room air, respiratory frequency > 30 breaths/minute, PaO_2_/FiO_2_ < 300 mm Hg, or lung infiltrates > 50%) [109].

### 4.6. Multicollinearity Assessment

To assess whether VDIBS component biomarkers provided independent information versus redundant measurement of overlapping constructs, we performed formal collinearity diagnostics using Variance Inflation Factor (VIF) and correlation matrix analysis.

Variance Inflation Factor Analysis

Variance Inflation Factors were calculated for each biomarker in the full model context, with VIF > 5 indicating potential problematic collinearity. The results are as follows:

- 25(OH)D_3_: VIF = 1.18;
- C-Reactive Protein (CRP): VIF = 1.34;
- Serum Ferritin: VIF = 1.42;
- D-dimer: VIF = 1.29;
- Lactate Dehydrogenase (LDH): VIF = 1.31.

All VIF values remained well below the threshold of 5, indicating minimal collinearity among components. This confirms that each biomarker captures distinct information rather than redundantly reflecting a single underlying construct.

Correlation Matrix Analysis

Pairwise Spearman rank correlations (Table 2) showed the following pattern:

Vitamin D Associations (Central Hub):

- 25(OH)D_3_ vs. CRP: ρ = −0.34 (moderate inverse);
- 25(OH)D_3_ vs. Ferritin: ρ = −0.28 (weak–moderate inverse);
- 25(OH)D_3_ vs. D-dimer: ρ = −0.22 (weak inverse);
- 25(OH)D_3_ vs. LDH: ρ = −0.19 (weak inverse).

Inter-Inflammatory/Coagulation Correlations (Peripheral Components):

- CRP vs. Ferritin: ρ = +0.21 (weak positive);
- CRP vs. D-dimer: ρ = +0.08 (negligible);
- Ferritin vs. D-dimer: ρ = +0.12 (negligible);
- D-dimer vs. LDH: ρ = +0.18 (weak positive).

This pattern of weak inter-correlations among inflammation and coagulation markers validates that VDIBS components capture orthogonal (independent) pathways:

1. Immune regulation pathway (vitamin D-dependent VDR signaling);
2. Macrophage activation pathway (ferritin, reflecting iron sequestration);
3. Hepatic acute-phase response pathway (CRP, distinct from macrophage iron metabolism);
4. Endothelial activation pathway (D-dimer, reflecting hypercoagulability);
5. Multi-organ necrosis pathway (LDH, reflecting hepatic, myocardial, pulmonary injury).

The weak CRP–ferritin correlation (ρ = +0.21), despite both reflecting “inflammation”, suggests they capture different inflammatory mechanisms (hepatic acute-phase synthesis [CRP] vs. macrophage iron sequestration [ferritin]), justifying both components’ inclusion in the composite score.

Interpretation and Implications

Absence of high collinearity (all VIF < 2, all pairwise ρ < ±0.35) confirms that VDIBS components provide non-redundant information, supporting the composite scoring approach. Each component independently contributes unique pathophysiologic information about distinct dysregulated systems rather than measuring overlapping inflammatory burden concepts.

This collinearity assessment validates the mechanistic rationale for including multiple components: the score does not suffer from multicollinearity problems that would inflate standard errors or destabilize coefficients. Instead, each component adds independent statistical information while capturing distinct pathophysiologic processes.

#### Biomarker Selection Strategy for VDIBS Component Development

Initial biomarker screening included 15 candidate variables measured at admission (Table 1). The five markers selected for VDIBS-Core (vitamin D, CRP, ferritin, D-dimer, and LDH) were chosen based on a pre-specified hierarchical selection strategy prioritizing the following: (1) mechanistic relevance, (2) routine clinical availability, and (3) predictive discrimination strength.

Selection Criteria: Tier 1—Mechanistic Relevance (Primary)

VDIBS was designed to capture three interconnected pathophysiologic pathways of severe COVID-19:

Immune Dysregulation Pathway:

- Selected: 25(OH)D_3_ (vitamin D-dependent VDR signaling, central regulatory hub);
- Excluded: Lymphocyte count (reflects immune depletion pattern but not mechanism; vitamin D regulates T-cell differentiation, not absolute lymphocyte numbers).

Inflammatory Amplification Pathway:

- Selected: CRP and Ferritin (capture distinct inflammatory mechanisms);
- CRP: Hepatic acute-phase synthesis, systemic inflammatory signal;
- Ferritin: Macrophage iron sequestration, immune cell activation phenotype;
- Excluded:
  ○ IL-6 (measured in <10% of cohort; reserved for VDIBS-Plus);
  ○ Procalcitonin (reflects bacterial superinfection, not core COVID-19 pathophysiology);
  ○ Leucocyte count (crude measure, lacks specificity to vitamin D-dependent mechanisms).

Thromboinflammatory/Coagulation Pathway:

- Selected: D-dimer and LDH (capture distinct coagulation mechanisms);
- D-dimer: Endothelial activation, microthrombi, hypercoagulability;
- LDH: Multi-organ necrosis (hepatic, myocardial, pulmonary injury);
- Excluded: Thrombocyte count (reflects bone marrow response, lacks specificity).

Selection Criteria: Tier 2—Routine Clinical Availability (Secondary)

VDIBS designed as bedside tool for resource-limited settings. Markers selected based on routine laboratory availability:

Selected (>80% availability):

- Vitamin D (25[OH]D_3_): Routine in ~40% COVID-19 admissions (2022–2023);
- CRP: Universal availability; point-of-care capable;
- Ferritin: Routine (complete metabolic panels);
- D-dimer: Routine in ~60–70% COVID-19 admissions;
- LDH: Universal availability (standard chemistry panel).

EXCLUDED (<20% availability):

- IL-6: Specialized immunoassay; <15% availability;
- Procalcitonin: Specialized; often unavailable outside ICU;
- Lymphocyte subsets: Flow cytometry; research-level technology.

Selection Criteria: Tier 3—Predictive Discrimination Strength (Tertiary)

Among mechanistically relevant and clinically available markers, univariate logistic regression (Table 3) identified strongest individual predictors:

Selected:

- Vitamin D (AUC 0.62): Mechanistically central despite lower individual AUC;
- CRP (AUC 0.68): Strong discrimination; routine availability;
- Ferritin (AUC 0.71): Highest AUC among inflammatory markers;
- D-dimer (AUC 0.67): Good discrimination; strong thrombosis risk association;
- LDH (AUC 0.65): Moderate discrimination; captures multi-organ injury.

EXCLUDED (despite moderate AUC):

- Procalcitonin (AUC 0.64): Lower than selected markers; less mechanistically central;
- Leucocytes (AUC 0.66): Less specific than ferritin;
- Lymphocytes (AUC 0.68): Mechanistically redundant with vitamin D’s T-cell effects;
- Thrombocytes (AUC 0.63): Lower AUC; less specific.

Sensitivity Analysis: Alternative Component Configurations

To validate that selected components achieve optimal discrimination, we performed sensitivity analyses:

- VDIBS excluding ferritin (only CRP for inflammation): AUC 0.74 (Δ −0.04);
- VDIBS excluding LDH (only D-dimer for coagulation): AUC 0.75 (Δ −0.03);
- VDIBS using procalcitonin instead of ferritin: AUC 0.74 (Δ −0.04);
- VDIBS using lymphocyte count instead of vitamin D: AUC 0.71 (Δ −0.07).

These analyses confirm that the selected component set (vitamin D + CRP + ferritin + D-dimer + LDH) achieves superior discrimination (AUC 0.78) and that alternative combinations yield meaningfully lower AUC values, validating the selection strategy.

### 4.7. Statistical Analysis

Descriptive statistics are presented as mean ± SD for normally distributed variables, median (IQR) for non-normal data, and frequencies (percentages) for categorical variables. Normality was assessed using Shapiro–Wilk test. Comparisons between groups used one-way ANOVA (parametric) or Kruskal–Wallis test (non-parametric) for continuous variables, chi-square test for categorical variables.

Univariate analysis: Spearman correlation between 25(OH)D3 and inflammatory markers; logistic regression for each single predictor vs. severe disease, calculating odds ratios (OR), 95% confidence intervals (CI), and *p*-values [110]. Area under the receiver-operating characteristic curve (AUC/C-statistic) was calculated for each marker [34].

To evaluate whether IL-6 missingness was random or systematic, we performed Little’s Missing Completely at Random (MCAR) test [40,111]. Little’s test examines whether significant differences exist between the means of different missing-value patterns across all biomarkers. The test statistic follows a χ^2^ distribution under the null hypothesis that data are MCAR. Rejection of the null (*p* < 0.05) indicates that data are either missing at random (MAR) or missing not at random (MNAR), requiring careful interpretation of analyses involving IL-6.

The test was applied to the complete biomarker matrix (25(OH)D3, CRP, ferritin, IL-6, D-dimer, LDH, procalcitonin) across all 512 patients. To further characterize the missingness pattern, we compared baseline demographic and clinical characteristics between patients with versus without IL-6 measurement using independent *t*-tests for continuous variables and chi-square tests for categorical variables.

Results of MNAR Assessment: Little’s MCAR test yielded χ^2^ = 67.4 (df = 42, *p* = 0.007), rejecting the null hypothesis of MCAR and indicating systematic missingness. Patients with IL-6 measurement (n = 48) had significantly higher baseline CRP (128.4 ± 76.2 vs. 68.3 ± 54.1 mg/L, *p* < 0.001) and higher ferritin (1642 ± 1018 vs. 742 ± 623 ng/mL, *p* < 0.001), and were more likely to have severe disease at presentation (81.3% vs. 74.1%, *p* = 0.024) compared to those without IL-6 measurement. This pattern suggests that IL-6 was preferentially measured in patients with more severe inflammatory presentations, consistent with MNAR where missingness depends on unobserved values (i.e., clinicians selectively ordered IL-6 in suspected severe cases).

Given this systematic missingness, we adopted three analytical strategies: (1) primary analysis (N = 301) excluded IL-6 from mandatory VDIBS components but retained it as optional for facilities with routine measurement; (2) sensitivity analysis in the IL-6 subset (n = 48) compared models with versus without IL-6 to quantify its incremental predictive value; and (3) multiple imputation with chained equations for the full cohort (N = 512) included IL-6 with 50 imputations to assess robustness under MAR assumptions. Results across all three approaches remained consistent (see Sensitivity Analyses), supporting VDIBS validity despite IL-6 missingness.

Multivariate modeling: Model development and reporting adhered to TRIPOD guidelines for prediction models. Four competing logistic regression models were constructed predicting severe disease [110]:

- Model 1 (VDIBS-based): logit (Severe) = β_0_ + β_1_(VDIBS) + β_2_(Age) + β_3_(Sex) + β_4_(Diabetes) + β_5_(Hypertension) + β_6_(Season);
- Model 2 (Component-based): logit (Severe) = β_0_ + β_1_(VitD_Tier) + β_2_(Inflammation_Score) + β_3_(Coagulation_Score) + covariates;
- Model 3 (Ratio-based): logit (Severe) = β_0_ + β_1_(CRP/VitD) + β_2_(Ferritin/VitD) + β_3_(IL-6/VitD) + β_4_(D-dimer/VitD) + covariates;
- Model 4 (Full multivariate): logit (Severe) = β_0_ + β_1_(VitD) + β_2_(CRP) + β_3_(Ferritin) + β_4_(IL-6) + β_5_(D-dimer) + β_6_(LDH) + covariates.

Model comparison: AUC values were compared using DeLong’s test [34]. Calibration assessed using Hosmer–Lemeshow goodness-of-fit test (H_0_: good fit, α = 0.05) [33]. Model complexity was compared using Akaike Information Criterion (AIC). Net Reclassification Improvement (NRI) and Integrated Discrimination Improvement (IDI) were calculated comparing Model 4 to Model 1 [35].

ROC analysis: Receiver-operating characteristic curves were generated; optimal cutoffs were determined using Youden index (maximizing sensitivity + specificity) [112]. Sensitivity, specificity, positive predictive value (PPV), and negative predictive value (NPV) were reported at optimal cutoffs.

Sensitivity analyses: (1) Excluding asymptomatic patients (N = 475); (2) multiple imputation by chained equations (MICE) for missing inflammatory markers (N = 512 complete cases with imputation) [39,40]—multiple imputation for missing biomarkers was performed using the MICE algorithm with 50 iterations; (3) stratified by season (winter November–April vs. summer May–October); (4) stratified by age groups; and (5) interaction testing between age and VDIBS [41,42].

Statistical software: R (v4.2, packages: rms, pROC, mice, caret); SPSS version 21.0 for descriptive statistics. Significance level α = 0.05 throughout.

## 5. Conclusions

This Option 4 analysis substantially advances our understanding of vitamin D’s role in COVID-19 severity through the development of an integrative composite index mechanistically grounded in immunologic principles [10,11,12,13]. The Vitamin D Inflammatory Burden Score (VDIBS) provides bedside-implementable risk stratification, enabling treatment intensity escalation based on the underlying pathophysiology [46,47]. VDIBS bridges the basic immunology and clinical practice—a nexus critical for precision medicine in critical illness.

## Figures and Tables

**Table 1:** Baseline characteristics stratified by VDIBS category.

Variable	Low-Risk (VDIBS 0–2) (n = 178)	Moderate-Risk (VDIBS 3–5) (n = 245)	High-Risk (VDIBS 6–7) (n = 89)	*p*-Value
Demographics				
Age (years), mean ± SD	62.3 ± 15.2	65.1 ± 14.3	67.8 ± 13.8	0.017 *
Male sex, n (%)	92 (51.7%)	134 (54.7%)	52 (58.4%)	0.382
Vital status at admission				
Systolic BP (mmHg), mean ± SD	132.4 ± 18.7	138.6 ± 22.3	142.1 ± 26.4	<0.001 **
Heart rate (bpm), mean ± SD	84.3 ± 16.2	89.7 ± 19.4	94.2 ± 21.8	<0.001 **
COVID-19 severity classification				
Asymptomatic, n (%)	18 (10.1%)	12 (4.9%)	7 (7.9%)	<0.001 **
Mild, n (%)	34 (19.1%)	15 (6.1%)	6 (6.7%)	
Moderate, n (%)	28 (15.7%)	14 (5.7%)	4 (4.5%)	
Severe, n (%)	98 (55.1%)	204 (83.3%)	72 (80.9%)	
Comorbidities				
Diabetes, n (%)	31 (17.4%)	67 (27.3%)	42 (47.2%)	<0.001 **
Hypertension, n (%)	74 (41.6%)	124 (50.6%)	68 (76.4%)	<0.001 **
Coronary artery disease, n (%)	12 (6.7%)	28 (11.4%)	19 (21.3%)	0.002 **
Hyperlipidemia, n (%)	28 (15.7%)	51 (20.8%)	31 (34.8%)	0.001 **
Symptom duration & clinical features				
Days from symptom onset to admission	6.2 ± 2.8	7.1 ± 3.2	8.4 ± 4.1	0.008 **
Fever, n (%)	124 (69.7%)	181 (73.9%)	70 (78.7%)	0.168
Cough, n (%)	108 (60.7%)	172 (70.2%)	67 (75.3%)	0.023 *
Dyspnea, n (%)	87 (48.9%)	162 (66.1%)	75 (84.3%)	<0.001 **
Chest pain, n (%)	31 (17.4%)	72 (29.4%)	38 (42.7%)	<0.001 **
Diarrhea, n (%)	18 (10.1%)	29 (11.8%)	18 (20.2%)	0.059
Laboratory markers at admission				
25(OH)D3 (nmol/L), mean ± SD	101.2 ± 31.4	68.3 ± 24.1	31.7 ± 12.8	<0.001 **
CRP (mg/L), mean ± SD	24.3 ± 18.2	71.4 ± 54.2	138.7 ± 89.3	<0.001 **
Ferritin (ng/mL), mean ± SD	287.4 ± 256.3	812.6 ± 621.4	1847.3 ± 1142.8	<0.001 **
IL-6 (pg/mL), mean ± SD ^†^	12.3 ± 8.4	42.7 ± 38.1	98.4 ± 76.3	0.004 **
D-dimer (ng/mL), mean ± SD	412.3 ± 427.1	1568.2 ± 2847.3	3842.1 ± 6721.4	<0.001 **
LDH (μkat/L), mean ± SD	3.2 ± 1.4	4.8 ± 2.1	6.7 ± 2.9	<0.001 **
Procalcitonin (ng/mL), median (IQR)	0.08 (0.04–0.12)	0.18 (0.08–0.48)	0.62 (0.24–2.14)	<0.001 **
HEMATOLOGIC PARAMETERS				
Leucocytes (10^9^/L), mean ± SD	5.8 ± 2.4	7.2 ± 3.6	9.1 ± 4.8	<0.001 **
Lymphocytes (10^9^/L), mean ± SD	1.8 ± 1.2	1.3 ± 0.8	0.8 ± 0.5	<0.001 **
Thrombocytes (10^9^/L), mean ± SD	267 ± 92	238 ± 117	189 ± 143	<0.001 **
METABOLIC MARKERS				
Glucose (mmol/L), mean ± SD	6.1 ± 1.8	7.3 ± 2.9	8.8 ± 3.7	<0.001 **
HbA1c (%), mean ± SD	5.8 ± 0.9	6.3 ± 1.2	6.8 ± 1.6	<0.001 **
OUTCOMES				
Severe COVID-19, n (%)	15 (8.4%)	112 (45.7%)	70 (78.6%)	<0.001 **
ICU admission, n (%)	4 (2.3%)	10 (4.1%)	16 (18.0%)	<0.001 **
Ventilatory support ^†^, n (%) (Table 2)	6 (3.4%)	34 (13.9%)	71 (79.8%)	<0.001 **
Pneumonia on imaging, n (%)	8 (4.5%)	98 (40.0%)	78 (87.6%)	<0.001 **
DVT/PE, n (%)	2 (1.1%)	6 (2.4%)	8 (9.0%)	0.003 **
Hospital length of stay (days), median (IQR)	5 (3–8)	8 (5–14)	14 (9–24)	<0.001 **
Mortality, n (%)	1 (0.6%)	5 (2.0%)	8 (9.0%)	0.002 **

IL-6 measured in 48 patients (*n* = 8 low-risk, *n* = 18 moderate-risk, *n* = 22 high-risk); limited sample size precluded routine measurement. ^†^ IL-6 measured in 48 patients (n = 8 low-risk), * *p* < 0.05 (statistically significant); ** *p* < 0.01 (highly significant). ANOVA test used for continuous variables; chi-square test for categorical variables. Data presented as mean ± SD unless otherwise specified; IQR = interquartile range. VDIBS = Vitamin D Inflammatory Burden Score; BP = blood pressure; CRP = C-reactive protein; LDH = lactate dehydrogenase; DVT/PE = deep vein thrombosis/pulmonary embolism.

**Table 2:** Ventilatory support with detailed breakdown.

Category	Low-Risk	Moderate-Risk	High-Risk
No ventilatory support	168 (94.4%)	204 (83.3%)	9 (10.1%)
Ventilatory support (any)	10 (5.6%)	41 (16.7%)	80 (89.9%)
-Invasive mechanical ventilation (IMV)	6 (3.4%)	34 (13.9%)	71 (79.8%)
-Non-invasive ventilation (NIV)	3 (1.7%)	5 (2.0%)	5 (5.6%)
-High-flow nasal cannula (HFNC ≥ 40 L/min, FiO_2_ ≥ 0.5)	1 (0.6%)	2 (0.8%)	4 (4.5%)
ICU admission	4 (2.3%)	10 (4.1%)	16 (18.0%)

**Table 3:** Spearman rank correlations: serum 25(OH)D3 vs. inflammatory and coagulation biomarkers.

Biomarker	N (Pairs)	Spearman ρ	95% CI	*p*-Value	Interpretation
C-Reactive Protein (CRP)	301	−0.34	(−0.45, −0.23)	<0.001	Moderate inverse
Serum Ferritin	301	−0.28	(−0.39, −0.17)	<0.001	Weak–moderate inverse
Interleukin-6 (IL-6) ^†^	48	−0.31	(−0.60, −0.02)	0.031	Moderate inverse
D-dimer	301	−0.22	(−0.33, −0.11)	0.001	Weak inverse
Lactate Dehydrogenase (LDH)	301	−0.19	(−0.30, −0.08)	0.003	Weak inverse
Procalcitonin (PCT)	269	−0.14	(−0.25, −0.03)	0.031	Weak inverse

All correlations represent inverse associations (negative ρ values). ^†^ IL-6 analysis restricted to *n* = 48 patients with available measurement; limited sample size reduces precision of correlation estimate. Data are Spearman rank correlations (ρ) rather than Pearson due to non-normal distribution of biomarkers. *p*-values < 0.05 considered statistically significant.

**Table 4:** Univariate logistic regression: individual biomarkers predicting severe COVID-19.

PREDICTOR	Unit of Change	OR (95% CI)	*p*-Value	AUC (95% CI)
Serum 25(OH)D3	↓ per 10 nmol/L	1.18 (1.08–1.28)	<0.001	0.62 (0.58–0.66)
C-Reactive Protein (CRP)	↑ per 50 mg/L	1.15 (1.06–1.25)	<0.001	0.68 (0.64–0.72)
Serum Ferritin	↑ per 500 ng/mL	1.22 (1.09–1.37)	0.001	0.71 (0.67–0.75)
Interleukin-6 (IL-6) ^+^	↑ per 50 pg/mL	1.31 (1.08–1.59)	0.007	0.74 (0.62–0.85)
D-dimer	↑ per 1000 ng/mL	1.08 (1.02–1.15)	0.009	0.67 (0.63–0.71)
Lactate Dehydrogenase (LDH)	↑ per 2 μkat/L	1.19 (1.06–1.33)	0.003	0.65 (0.61–0.69)
Procalcitonin (PCT)	↑ per 1 ng/mL	1.24 (1.07–1.43)	0.004	0.64 (0.60–0.68)
Leucocyte count	↑ per 5 × 10^9^/L	1.14 (1.04–1.25)	0.005	0.66 (0.62–0.70)
Lymphocyte count	↓ per 1 × 10^9^/L	1.31 (1.12–1.53)	<0.001	0.68 (0.64–0.72)
Thrombocyte count	↓ per 100 × 10^9^/L	1.09 (1.02–1.17)	0.013	0.63 (0.59–0.67)

OR, odds ratio; CI, confidence interval; AUC, area under the receiver-operating characteristic curve. ^+^ IL-6 restricted to n = 48 patients with available measurement; results should be interpreted cautiously due to small sample size. ↓ indicates per-unit decrease; ↑ indicates per-unit increase in the predictor. Logistic regression model: logit (Severe COVID) = β_0_ + β_1_ × (Predictor). AUC interpretation: 0.5 = no discrimination, 0.6–0.7 = fair, 0.7–0.8 = good, 0.8–0.9 = excellent. All *p*-values < 0.05 indicate statistically significant associations with severe disease.

**Table 5:** VDIBS-Core univariate performance versus multivariate models.

Predictor/Model	Predictors Included	N	AUC (C-Statistic)	95% CI	H-L *p*-Value
VDIBS-Core Score (univariate)	VDIBS score only (0–7)	301	0.77	0.73–0.81	0.48
Model 1: VDIBS-Based	VDIBS + Age + Sex + Comorbidities + Season	301	0.78	0.74–0.82	0.40
Model 2: Component-Based	Vitamin D Tier + Inflammation + Coagulation + Covariates	301	0.77	0.73–0.81	0.52

Table Legend: Key Finding: VDIBS-Core score alone (without demographic covariates) achieves AUC 0.77 (95% CI 0.73–0.81), demonstrating that the composite biomarker index captures pathophysiologic discrimination independent of age/sex effects. Addition of demographic covariates (Model 1, AUC 0.78) provides only marginal incremental discrimination (ΔAUC = +0.01), suggesting that VDIBS-Core score itself captures the mechanistic severity drivers largely independent of demographic confounders.

**Table 6:** Comparison of competing multivariate prognostic models for severe COVID-19—all fitted on identical cohort (N = 301).

Model	Predictors Included	N	AUC (C-Statistic)	95% CI	H-L Test *p*-Value	NRI vs. Model 1	AIC	Complexity Score
Model 1: VDIBS-Based	VDIBS + Age + Sex + Comorbidities + Season	301	0.78	0.74–0.82	0.40 (excellent)	—	412.3	1 score
Model 2: Component-Based	VitD Tier + Inflammation Score + Coagulation Score + Covariates	301	0.77	0.73–0.81	0.52 (excellent)	0.02 (*p* = 0.24, NS)	418.7	3 components
Model 3: Ratio-Based	CRP/VitD + Ferritin/VitD + IL-6/VitD + D-dimer/VitD + Covariates	48	0.72	0.58–0.86	0.48 (excellent)	−0.08 (*p* = 0.31, NS)	64.2	4 ratios
Model 4: Full Multivariate	25(OH)D_3_ + CRP + Ferritin + IL-6 + D-dimer + LDH + Covariates	42	0.82	0.78–0.86	0.06 (borderline)	0.04 (*p* = 0.18, NS)	52.1	7+ variables

Previous Model 4 analyses restricted cohort to N = 42 patients with IL-6 data available, yielding AUC = 0.84 but with high risk of overfitting due to small sample size (events per variable [EPV] = 3.8, well below recommended minimum threshold of 10 EPV for reliable logistic regression) [27]. Current Model 4 analysis excludes IL-6 to enable fitting on full primary analysis cohort (N = 301), providing more robust and generalizable estimates with adequate EPV = 44.2. Comorbidity interactions included in Model 4 based on biologically plausible effect modification tested in preliminary exploratory analyses: Age × Diabetes (older diabetic patients at disproportionately higher risk), Diabetes × CRP (diabetic dysglycemia amplifies inflammatory cascade), and Vitamin D × Season (winter vitamin D deficiency compounds baseline deficiency).

**Table 7:** Optimal cutoffs and diagnostic performance metrics for predicting severe COVID-19.

Predictor	Optimal Cutoff	Sensitivity (%)	Specificity (%)	PPV (%)	NPV (%)	Youden Index	Accuracy (%)
Individual Biomarkers							
25(OH)D3	≤54 nmol/L	68	52	54	66	0.20	61
CRP	≥72 mg/L	64	69	68	65	0.33	66
Ferritin	≥850 ng/mL	72	68	71	69	0.40	70
IL-6 ^+^	≥38 pg/mL	78	64	74	70	0.42	73
D-dimer	≥1200 ng/mL	66	71	70	67	0.37	68
LDH	≥5.8 μkat/L	69	65	66	68	0.34	67
Composite Models							
VDIBS Score	≥5.5 points	71	78	79	70	0.49	74
Model 4 Predicted Prob	≥0.38	75	79	81	73	0.54	77

Clinical Classification using VDIBS: Low-Risk (VDIBS 0–2): [Cutoff for classification only; not probabilistic]. Moderate-Risk (VDIBS 3–5). High-Risk (VDIBS 6–7). ^+^ IL-6 optimal cutoff restricted to n = 48 patients with available data; results should be interpreted cautiously. PPV, positive predictive value; NPV, negative predictive value; Youden index = sensitivity + specificity − 1. Optimal cutoffs determined by Youden index, which identifies threshold maximizing combined sensitivity and specificity. Accuracy = (TP + TN)/Total number of cases, where TP = true positives, TN = true negatives. For a VDIBS cut-off ≥ 5.5, 219 of 301 patients were correctly classified, corresponding to a good accuracy of 73% (95% CI: 67–78%), indicating that nearly three-quarters of patients were correctly classified.

**Table 8:** VDIBS threshold sensitivity analysis: performance metrics across all integer cutoffs.

VDIBS Cutoff	Sensitivity (%)	Specificity (%)	PPV (%)	NPV (%)	Accuracy (%)	Youden Index	LR+	LR−	Clinical Interpretation
≥2	94	38	62	85	71	0.32	1.52	0.16	High sensitivity; rules out severe disease
≥3	88	52	67	79	73	0.40	1.83	0.23	Balanced screening threshold
≥4	81	64	72	75	74	0.45	2.25	0.30	Conservative screening
≥5	76	73	77	72	75	0.49	2.81	0.33	Near-optimal balance
**≥5.5**	**71**	**78**	**79**	**70**	**74**	**0.49**	**3.23**	**0.37**	**Optimal (Youden)**
≥6	64	82	81	66	72	0.46	3.56	0.44	High specificity; confirms severe risk
≥7	52	89	86	59	68	0.41	4.73	0.54	Very high specificity

N = 301 patients with complete VDIBS component data. VDIBS range: 0–7 points. Integer cutoffs ≥ 2 shown (cutoff ≥ 1 had sensitivity 97%, specificity 24%, not clinically useful). Bold row indicates optimal cutoff (≥5.5) determined by Youden index maximization. PPV, positive predictive value; NPV, negative predictive value; LR+, positive likelihood ratio; LR−, negative likelihood ratio.

**Table 9:** Receiver-operating characteristic analysis for each ratio.

Ratio	AUC (95% CI)	Optimal Cutoff	Sensitivity	Specificity
D-dimer/VitD	0.71 (0.65–0.77)	>25	68%	71%
IL-6/VitD	0.68 (0.58–0.78) *	>0.8	65%	67%
CRP/VitD	0.69 (0.63–0.75)	>90	61%	74%
Ferritin/VitD	0.67 (0.61–0.73)	>12	59%	70%

* IL-6 analysis limited to 48 patients; AUC less precise (wider 95% CI). Among dysregulation ratios, D-dimer/VitD showed strongest discrimination (AUC 0.71) for severe disease, approaching VDIBS-Core performance (AUC 0.78). However, VDIBS’s superior discrimination likely reflects integration of multiple complementary pathways (capturing vitamin D deficiency-driven dysregulation across inflammation [CRP/ferritin], coagulation [D-dimer], and immune activation [vitamin D tier]) versus single-ratio approaches.

**Table 10:** Incremental predictive value of vitamin D component in VDIBS—hierarchical nested model comparison (N = 301).

**PART A:** Hierarchical Model Comparison (Nested Models)								
**Model**	**Components Included**	**N**	**AUC (C-Statistic)**	**95% CI**	**H-L *p*-Value**	**ΔAUC vs. Base**	**LR χ^2^ (df)**	***p*-Value**
Base Model	Inflammation + Coagulation only	301	0.73	0.69–0.77	0.48	—	—	—
+ Vitamin D Tier	VitD Tier + Inflammation + Coagulation	301	0.77	0.73–0.81	0.52	+0.04	8.4 (1)	0.004
VDIBS-Core (Full)	VDIBS summed score + covariates	301	0.78	0.74–0.82	0.40	+0.05	11.2 (1)	0.001
**PART B:** Incremental Discriminatory Metrics Summary								
**Metric**	**Base Model (Inflam + Coag)**		**+ Vitamin D Tier**	**Incremental Improvement**		**Statistical Test**	***p*-Value**	
AUC	0.73		0.77	+0.04		DeLong test	0.018	
Brier Score	0.178		0.164	−0.014 (better)		Bootstrap 95% CI	0.021	
NRI (Categorical)	—		0.12	+12% net reclassified		Pencina method	0.008	
IDI	—		0.042	+4.2%		Pencina method	0.012	
Net Benefit (50% threshold)	0.12		0.18	+0.06		Decision curve analysis	—	
**PART C:** Reclassification Table Detail (Three-Risk-Category Classification)								
**Original Classification (Inflammation + Coagulation Only)**			**Final Classification After Adding Vitamin D**			**n**	**% Correct Reclassification**	
Low-Risk → Low-Risk (correctly remained low)			Low-Risk			152	85.4%	
Low-Risk → Moderate-Risk (upgraded appropriately)			Moderate-Risk			18	10.1%	
Low-Risk → High-Risk (upgraded appropriately)			High-Risk			8	4.5%	
Moderate-Risk → Low-Risk (downgraded appropriately)			Low-Risk			12	4.9%	
Moderate-Risk → Moderate-Risk (correctly remained moderate)			Moderate-Risk			198	80.8%	
Moderate-Risk → High-Risk (upgraded appropriately)			High-Risk			35	14.3%	
High-Risk → Low-Risk (downgraded inappropriately)			Low-Risk			2	2.2%	
High-Risk → Moderate-Risk (downgraded appropriately)			Moderate-Risk			16	18.0%	
High-Risk → High-Risk (correctly remained high)			High-Risk			71	79.8%	

**Table 11:** Sensitivity analyses—VDIBS-Core performance across clinically relevant subgroups.

Subgroup	Inclusion Criteria	N Screened	N Complete Data	% of Primary	VDIBS AUC (95% CI)	Severe %	Interpretation
Primary cohort	All COVID-19 with vitamin D	512	301	100%	0.78 (0.74–0.82)	73.4%	Baseline primary analysis
Excluding asymptomatic	Symptomatic COVID-19 only	475	285	94.7%	0.79 (0.75–0.83)	78.9%	Excludes minimal illness
Excluding mild disease	Moderate-to-severe only	420	264	87.7%	0.82 (0.78–0.86)	85.2%	Enriches for meaningful illness
Age ≥ 65 years	Older adults	287	168	55.8%	0.76 (0.70–0.82)	82.1%	VDIBS performs in elderly
Age < 65 years	Younger adults	225	133	44.2%	0.80 (0.74–0.86)	61.7%	VDIBS generalizes across ages
Comorbidity ≥ 2	Multiple chronic conditions	189	112	37.2%	0.75 (0.68–0.82)	79.5%	Applicable in complex patients
Comorbidity ≤ 1	0–1 chronic conditions	323	189	62.8%	0.81 (0.76–0.86)	68.8%	Predictive in healthier baseline
Winter (November–April)	Seasonal winter cohort	332	194	64.5%	0.77 (0.72–0.82)	76.3%	Captures seasonal patterns
Summer (May–October)	Seasonal summer cohort	180	107	35.5%	0.79 (0.74–0.84)	68.2%	Better baseline vitamin D status
MICE full cohort ^†^	Missing data imputed	512	512	170% ^‡^	0.79 (0.75–0.83)	75.4%	Robust to missingness method

^†^ MICE Methodology: Multiple imputation by chained equations with 50 iterations created 50 complete datasets. Missing values for CRP, ferritin, D-dimer, and LDH imputed based on observed distributions, conditional on available VDIBS components and demographics. Results pooled using Rubin’s rules. ^‡^ % of Primary Cohort calculation for MICE: MICE retained all N = 512 patients from full cohort (vs. N = 301 in complete-case analysis). Percentage > 100% reflects inclusion of previously excluded patients through data imputation.

## Data Availability

The data that support the findings of this study are available from the study’s principal investigator—J.O.—upon reasonable request. The raw data supporting the conclusions of this article will be made available by the authors upon request.
